# A Putative Cation Channel, NCA-1, and a Novel Protein, UNC-80, Transmit Neuronal Activity in C. elegans

**DOI:** 10.1371/journal.pbio.0060055

**Published:** 2008-03-11

**Authors:** Edward Yeh, Sharon Ng, Mi Zhang, Magali Bouhours, Ying Wang, Min Wang, Wesley Hung, Kyota Aoyagi, Katya Melnik-Martinez, Michelle Li, Fang Liu, William R Schafer, Mei Zhen

**Affiliations:** 1 Samuel Lunenfeld Research Institute, Mount Sinai Hospital, Toronto, Ontario, Canada; 2 Department of Molecular Genetics, University of Toronto, Toronto, Ontario, Canada; 3 Division of Biology, University of California San Diego, San Diego, California, United States of America; 4 Department of Neuroscience, Centre for Addiction and Mental Health, Clarke Division, University of Toronto, Toronto, Ontario, Canada; 5 Cell Biology Division, MRC Laboratory of Molecular Biology, Cambridge, United Kingdom; Cambridge University, United Kingdom

## Abstract

Voltage-gated cation channels regulate neuronal excitability through selective ion flux. NALCN, a member of a protein family that is structurally related to the α1 subunits of voltage-gated sodium/calcium channels, was recently shown to regulate the resting membrane potentials by mediating sodium leak and the firing of mouse neurons. We identified a role for the Caenorhabditis elegans NALCN homologues NCA-1 and NCA-2 in the propagation of neuronal activity from cell bodies to synapses. Loss of NCA activities leads to reduced synaptic transmission at neuromuscular junctions and frequent halting in locomotion. In vivo calcium imaging experiments further indicate that while calcium influx in the cell bodies of egg-laying motorneurons is unaffected by altered NCA activity, synaptic calcium transients are significantly reduced in *nca* loss-of-function mutants and increased in *nca* gain-of-function mutants. NCA-1 localizes along axons and is enriched at nonsynaptic regions. Its localization and function depend on UNC-79, and UNC-80, a novel conserved protein that is also enriched at nonsynaptic regions. We propose that NCA-1 and UNC-80 regulate neuronal activity at least in part by transmitting depolarization signals to synapses in C. elegans neurons.

## Author Summary

Neurons communicate to their targets through synapses that are activated by the electrical signals conveyed along neuronal processes. The tightly regulated ion flux across the cell membrane drives the generation of these electrical signals; it is therefore important to identify ion channels that regulate the excitability of neurons. In the C. elegans nervous system, we reveal that a putative channel complex, consisting of ion-conducting, pore-forming proteins called NCAs and two auxiliary components called UNC-79 and UNC-80, regulates neuronal function. We first show that an increase or decrease of the activity of this channel causes physiological changes that indicate corresponding alterations in neuronal activity. We then demonstrate by in vivo calcium imaging that the NCA channel, localizing along axons, specifically regulates excitation of synapses. We speculate that this channel participates in the propagation of electric signals that activate synapses.

## Abstract 3

A putative cation channel NCA/UNC-79/UNC-80 modulates the propagation of excitation signals along axons in *C. elegans* neurons. Components conserved throughout evolution, these channel complexes may play similar roles in other nervous systems.

## Introduction

Neurons generate and propagate electrical signals along nerve processes, which are converted into chemical communication through neurotransmitter release at synapses. By allowing selective ion flux across the plasma membrane, cation channels regulate the excitation and function of neurons. In most nervous systems, action potentials, the traveling and rapidly reversing membrane potentials, are induced by the opening of voltage-gated sodium channels and are modulated by voltage-gated sodium, potassium, and occasionally calcium (Ca^2+^) channels [1,2]. Action potential–induced depolarization at presynaptic termini triggers the opening of voltage-gated calcium channels (VGCCs), leading to an influx of Ca^2+^ that allows for Ca^2+^-dependent synaptic vesicle exocytosis and the release of neurotransmitters [3].

Voltage-gated sodium channels consist of a pore-forming α1 subunit and variable numbers of auxiliary β subunits [4]. They display similar properties and have similar functions in establishing membrane thresholds, and generating and propagating action potentials. In contrast, multiple neuronal VGCCs differ in composition, property, localization, and function. All known VGCCs are composed of a pore-forming α1 subunit, which associates with various accessory α2δ, β, and γ subunits that modulate the property of the channel [4–6]. Vertebrates have at least six subfamilies of VGCCs with different opening probabilities and kinetics [4–6]. Among them, P/Q- and N-type VGCCs are components of the active zone, the presynaptic subcellular structure where synaptic vesicles are released [7,8]. They mediate the Ca^2+^ influx that triggers the membrane fusion between synaptic vesicles and presynaptic termini [9]. Other VGCCs can also participate in the modulation of neuronal excitation, affecting the duration of action potentials of specific neurons [2,10].

C. elegans does not encode voltage-gated sodium channel orthologues or display typical voltage-gated sodium currents [11–16]. Therefore, C. elegans cells either do not have action potentials, or generate and propagate atypical action potentials through alternative mechanisms such as VGCCs in muscles [13,15,17]. In C. elegans neurons, the nature of the excitation signals that lead to the depolarization at synapses, and how they are transmitted, are unknown. It was proposed that their membrane properties allow the passive spreading of electrical signals along axons in the sensory neurons [12]. Alternatively, they may also generate atypical action potentials.

C. elegans encodes a single P/Q-, N-, and R-family VGCC α1 subunit (UNC-2), one L-type α1 subunit (EGL-19), and one T-type α1 subunit (CCA-1) [17–19]. UNC-2 is proposed to localize at presynaptic active zones and affects neurotransmitter release [20]. The loss of UNC-2 function leads to slow and abnormal locomotion, failure in neuronal migration, and abnormal sensitivity to dopamine and serotonin [19–21]. In pharyngeal muscles, the excitability threshold is set by CCA-1, which initiates an atypical action potential in response to depolarization [15,17]. EGL-19 generates Ca^2+^ transients that define sarcomere excitability [13,15,18]. It also contributes to the Ca^2+^ transients in cultured mechanosensory neuron cell bodies [22].

A rat cDNA clone encoding a protein with homology to the α1 subunit of voltage-gated calcium and sodium channels was first isolated by a degenerative oligo-based PCR screening [23]. Homologues of this protein are present in various animals, namely NCA-1 and NCA-2 in C. elegans [24], Dmα1U/CG1517 in *Drosophila* [25], and Vgcnl1/NALCN [23,26] in mouse, rat, and human. Unlike all known sodium and calcium channel α1 subunits, whose ion selectivity filter motifs are DEKA [27] and EEEE [28,29], respectively, these proteins contain EEKE at corresponding positions. They also display divergence from the known voltage-gated sodium/calcium channels by a reduction of charged amino acids in the voltage-sensing fourth transmembrane domains, suggesting that they may form channels with unique properties. Indeed, a recent paper showed that the rat NALCN forms a voltage-insensitive and poorly selective cation leak channel in HEK293T cells [26].

*Drosophila Dmα1U* mutants are viable but display altered sensitivity to anesthetics and abnormal circadian rhythm [25]. *C. elegans nca-1;nca-2* double knockout mutants also display abnormal halothane sensitivity and more frequent pauses during locomotion, a phenotype termed “fainter” [24]. The physiological basis for these defects, however, is unknown. *NALCN* knockout mice are neonatal lethal due to a disrupted respiratory rhythm [26]. Mutant hippocampal neurons display reduced background Na^+^ leak currents and decreased firing, suggesting that NALCN functions as a Na^+^ leak channel and regulates neuronal excitability by affecting membrane potentials [26].

In this study, we describe the physiological and cell biological characterization of the NCA proteins in C. elegans. Our genetic and phenotypic analyses of *nca* loss- and gain-of-function mutants show that NCA proteins affect synaptic function by modulating the transmission of depolarization signals. This function depends on two novel auxiliary proteins: UNC-79 and UNC-80. Thus, a putative NCA channel regulates neuronal activity in C. elegans neurons, at least in part by facilitating axonal conductance of depolarizing signals from the cell body to the synapse.

## Results

### NCA Is Required for Synaptic Transmission at GABAergic and Cholinergic Neuromuscular Junctions (NMJs)

**Figure 1:**
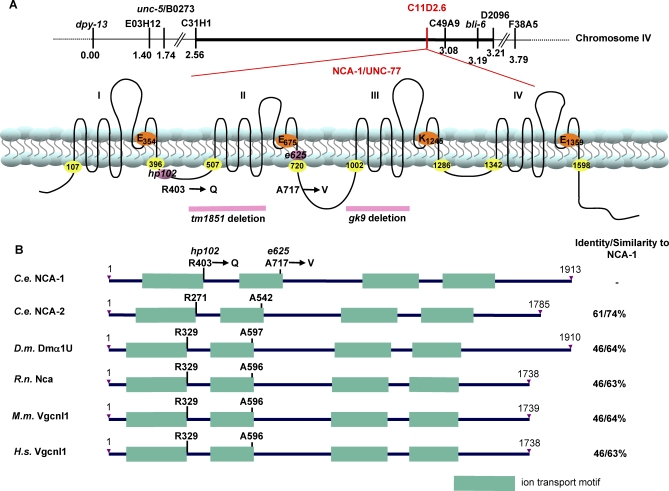
*hp102* and *e625* Encode NCA-1, a Cation Channel α_1_-Like Subunit **(**A**)** Genetic mapping of *nca(gf)* mutants and a schematic representation of the *nca-1* genetic locus (top) and the predicted NCA-1 protein structure (bottom). The positions of the *nca(gf)* (*hp102* and *e625*) and *nca(lf)* mutations (*gk9* and *tm1851*) are illustrated. Numbers with yellow highlights denote the beginning and end of each ion transport motif. The amino acid residues that determine the ion selectivity in related cation channels are highlighted with orange circles. **(**B) Similarity between NCA family members. The residues equivalent to *hp102* and *e625* are indicated. *C.e.*: C. elegans; *D.m.: D. melanogaster*; *R.n.: R. norvagicus*; *M.m.: M. musculus*; *H.s*.: *H. sapiens*.

To investigate the function of NCA channels in C. elegans, we identified and analyzed the phenotypes of animals carrying dominant and recessive mutations in *nca-1* and its homologue, *nca-2*. Both dominant and recessive mutations in NCAs have clear effects on C. elegans behavior. *gk9* and *gk5*, the two deletion alleles for *nca-1* and *nca-2*, respectively, were generated by the C. elegans gene knockout consortium. Removing part of the essential pore-forming domain of NCA-1 and NCA-2, both mutations are predicted to cause severe losses of protein functions (Figure 1A). While either single deletion mutants display normal locomotion, *nca-1(gk9);nca-2(gk5)* double mutants are fainters that fail to sustain sinusoidal locomotion and succumb to long periods of halting ([24,30], Videos S1 and S2). The fainter phenotype of *gk9;gk5* mutants is recessive and fully penetrant. This synergism, together with our results presented in later sections, suggest that the phenotypes of *gk9;gk5* mutants represent the physiological outcome of the complete loss of NCA activity, which we will henceforth refer to as *nca(lf)*.

We identified two gain-of-function alleles of *nca-1* (see Materials and Methods, Figure 1A). One of these mutants, *hp102*, was isolated in a screen for developmental defects in active zone markers in GABAergic neurons [31] (Figure 1A); whereas the other allele, *e625*, was isolated as a locomotion-abnormal mutant originally named *unc-77* [32]. We identified a single missense mutation that alters residues at positions flanking IS6, the sixth transmembrane domain in the first repeat (R403Q), or within IIS6, the sixth transmembrane domain of the second repeat (A717V) of NCA-1 in *hp102* and *e625* mutants, respectively. Both affected amino acids are conserved in the protein family (Figure 1B). Unlike the recessive fainter phenotype of *nca(lf)* (Video S2), both *hp102* and *e625* showed semi-dominant, uncoordinated, and exaggerated body bends during either spontaneous or stimulated locomotion (referred to as “coiler” phenotype henceforth) (Video S3). Moreover, the expression of a *nca-1* genomic fragment that harbors the *hp102* mutation in wild-type animals induced locomotion defects similar to that in *hp102* mutants (Video S4, see Materials and Methods). In summary, both *hp102* and *e625* represent *nca-1* gain-of-function alleles, which may induce elevated, misregulated, or altered NCA activities. They will henceforth be referred to as *nca(gf)*.

**Figure 2:**
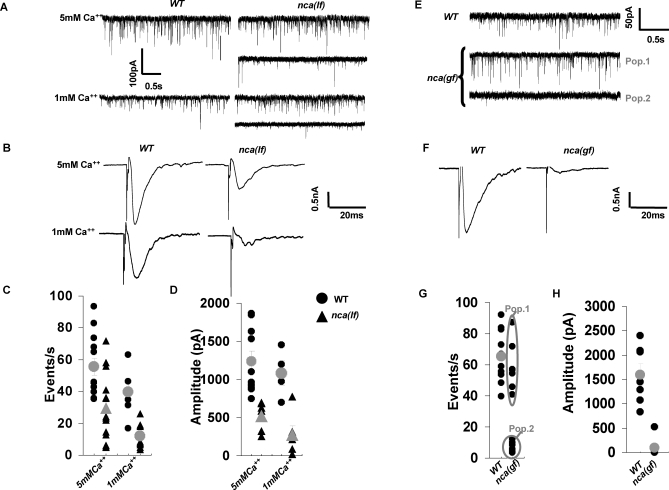
NCA-1 Activity Is Required for Normal Synaptic Transmission at NMJs Representative traces of spontaneous activity [(A) for *nca(lf)* and (E) for *nca(gf)*] and responses evoked in muscle by an electric stimulation of the ventral nerve cord [(B) for *nca(lf)* and (F) for *nca(gf)*] are shown. (A–D) The *nca(lf)* mutant mPSCs varied between wild-type (WT) values (*nca(lf)*, upper trace) and decreased frequency and amplitude (*nca(lf)*, lower trace). The overall mPSC frequency (C) and evoked response amplitude (D) were decreased in *nca(lf)* animals as compared to wild-type at either 5 mM (*nca(lf)*: *n* = 15; WT: *n* = 13) or 1 mM (*nca(lf)*: *n* = 9; WT: *n* = 6) extracellular Ca^2+^. No significant change in mPSC amplitude and distribution was observed between *nca(lf)* and WT animals. Individual results are shown in black, mean ± SEM in gray, for recordings at 5 mM and 1 mM extracellular Ca^2+^. (E–H) Representative traces of spontaneous (E) and evoked (F) post-synaptic currents at the neuromuscular junction at 5 mM Ca^2+^. The *nca(gf)* mutant mPSCs were either comparable to WT (population 1) or highly decreased in frequency and amplitude (population 2), whereas highly reduced (if any) responses could be evoked by electric stimulation of the nerve cord (F). The mPSC frequency (G) and evoked responses amplitude (H) are plotted for N2 and *nca(gf)* animals. Individual results are shown in black, mean ± SEM in gray, when relevant. (WT, mPSCP, *n* = 10, ePSP, *n* = 7; *nca(gf),* mPSP, *n* = 13, ePSP, *n* = 8) Error bars: SEM, *p ≤ 0.05, **p < 0.01. Statistic analysis was performed with Student's *t*-test.

The locomotion defects of both *nca(lf)* and *nca(gf)* mutants suggest that NCA activity regulates synapse function. To address this possibility, we recorded spontaneous and evoked postsynaptic currents in body wall muscles as an indirect measure for presynaptic activities of GABAergic and cholinergic neurons NMJs [33,34]. In the presence of both high and low concentrations of extracellular Ca^2+^, *nca(lf)* mutants displayed a significant decrease in the frequency of spontaneous release (miniature postsynaptic current, mPSC) (29.4 ± 5.3 Hz, *p* < 0.01 at 5mM Ca^2+^ and 11.8 ± 2.5 Hz, *p* < 0.001 at 1mM Ca^2+^) as compared to wild-type animals (55.6 ± 5.3 Hz at 5mM Ca^2+^ and 39.7 ± 6.5 Hz at 1mM Ca^2+^) (Figure 2A and 2C). They also displayed significantly reduced evoked responses. Electric stimulation of the ventral nerve cord in wild-type animals elicited currents (evoked postsynaptic current, ePSC) of 1234.1 ± 57.7 pA in amplitude at 5mM Ca^2+^, and 1080 ± 161.3 pA at 1mM Ca^2+^ (Figure 2B and 2D). In *nca(lf)* mutants, the amplitude of ePSC was reduced by 60% at 5mM Ca^2+^ (523.9 ± 57.7 pA, *p* < 0.001), and by 75% at 1 mM Ca^2+^ (278.6 ± 109.2 pA, *p* = 0.01) (Figure 2B and 2D). The decreased mPSC frequency and ePSC amplitude suggest a reduction of synaptic transmission at NMJs in *nca(lf)* mutants.

We also examined how *nca(gf)* mutations affect synaptic transmission. At 5 mM extracellular Ca^2+^, some *nca(gf)* animals (Figure 2E and 2G, population 1) displayed normal frequency of mPSC (59.1 ± 6.0 pA, *n* = 7 versus 65.4 ± 5.4 pA, *n* = 10 for wild-type), others (Figure 2E and 2G, population 2) had no mPSC at all (7.2 ± 1.4 pA, *n* = 6). No ePSC could be evoked in any of the two groups (Figure 2F and 2H). Although the cause of these abnormalities was not clear, these results indicate that *nca(gf)* animals also show aberrant synaptic activity and further establish the link between NCA channels and synaptic function.

### NCA Activity Regulates Presynaptic Activation at Serotonergic NMJs

**Figure 3:**
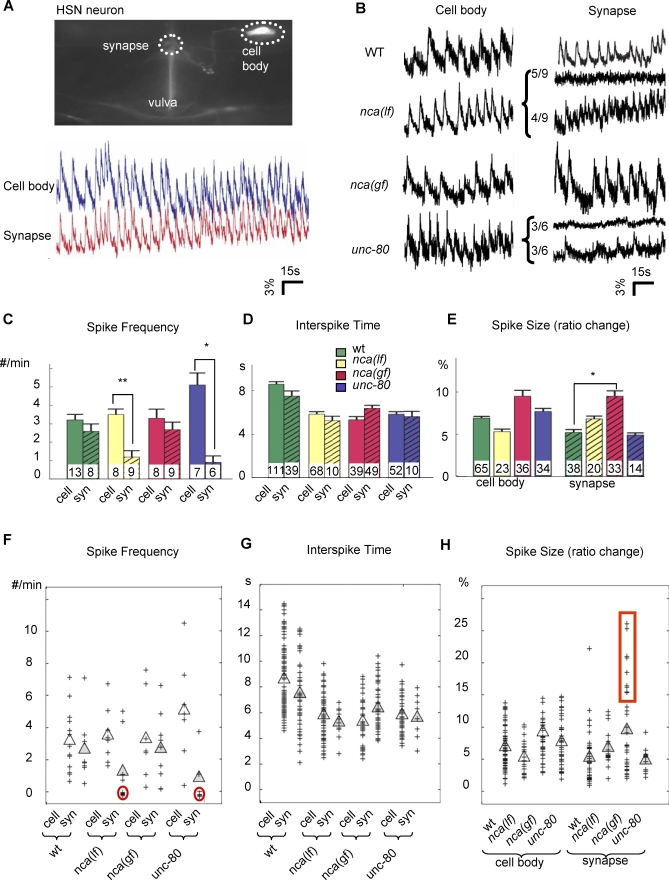
Ca^2+^ Transients at HSN Cell Bodies and Synapses in *nca(lf)*, *nca(gf)*, and *unc-80* Mutants (A) Upper panel: Image of HSN neuron used in calcium imaging. The HSN cell body and the synapse where Ca^2+^ imaging were performed are circled in dots. Lower panel: Sample traces of simultaneous recording of calcium spikes of both cell body and synapse, showing the synchronicity of the calcium signals. (B) Sample traces of yellow/cyan ratio that represent the relative Ca^2+^ concentration in HSN cell bodies (left panels) or their synapses (right panels). *x*-axis: time in s; *y*-axis: yellow/cyan ratio in percent. For *nca(lf*) and *unc-80*, animals displayed traces with either silent (top) or active (bottom) Ca^2+^ transients. (C–E) Histograms for the total spike frequency, spike interval, and size of calcium spikes detected in each strain. Arrowheads: examples of calcium spikes. The number of animals examined, *n,* (C) or the number of calcium spikes examined (D, E), is illustrated at the bottom of each bar. Error bar: SEM. *p < 0.05, **p < 0.005. (C) Average number of spikes/min for each genotype. There is no statistically significant difference for HSN cell bodies (cell) among all strains, or between HSN cell bodies (cell) and synapses (syn) of the same strain except for *nca(lf)* and *unc-80.* (D) Average time interval between two consecutive spikes within trains of calcium transients. There is no statistically significant difference between HSN cell bodies and corresponding synapses for all strains. (E) Average spike size. Wild-type, *nca(lf)*, and *unc-80* neurons displayed no statistically significant difference in spike size, but for the *nca-1(gf)* neurons, it was increased. (F–H) Scatter plots for spike frequency, interspike time interval, and spike size. Each cross represents a data point. Clear and filled triangles represent mean numbers for cell body and synapse calcium transients, respectively. Red circles highlight populations of animals with silenced synapse calcium transients. Red box highlights a population of *nca(gf)* synapse spikes that were significantly larger than those seen in other genotypes. All statistic analysis was performed by Kolmogorov-Smirnov rank test.

To investigate how altered NCA activity regulates presynaptic function, we examined neuronal excitation directly with cameleon, a genetically encoded Ca^2+^ sensor, in live C. elegans [35]. We focused on the serotonergic HSN motoneurons, where we also observed both morphological (abnormal active zone marker distribution) and behavioral (constitutive egg-laying) defects associated with their synapses in *nca(gf)* mutants (Figure S1D–S1F). Most importantly, the unusually large size of HSN synapses provided us the unique opportunity to perform in vivo simultaneous Ca^2+^ imaging at both soma and the presynaptic regions (Figure 3A).

When C. elegans is immersed in solutions that constitutively activate egg-laying (see Materials and Methods), HSNs—the motoneurons that innervate the egg-laying vulval muscles—autonomously initiate periodic trains of Ca^2+^ transients in cell bodies that are independent of presynaptic inputs (M. Zhang et al., unpublished data, Figures S2 and S3, Video S5). These transients temporally correlated with the Ca^2+^ spikes in the presynaptic region (Figure 3A, blue and red traces; Figures S2 and S3). The Ca^2+^ transients at presynaptic regions and cell bodies displayed similar spike frequency (2.6 ± 0.8 spikes/min at synapses versus 3.2 ± 0.6 spikes/min at cell bodies, *p* > 0.05, Figure 3C and 3F) and similar time intervals between spikes in the trains (7.5 ± 0.5 s at synapses versus 8.6 ± 0.2 s at cell bodies, *p* > 0.05, Figure 3D and 3G), suggesting that the depolarization signals were generated at the cell bodies and quickly spread to the presynaptic regions.

Under the same conditions, in *nca(lf)* mutants, HSN cell bodies generated trains of Ca^2+^ spikes undistinguishable from those in wild-type soma for spike frequency (3.5 ± 0.6 spikes/min versus 3.2 ± 0.6 spikes/min for wild-type, *p* > 0.05), interval (5.8 ± 0.2 s versus 8.6 ± 0.2 s for wild-type, *p* > 0.05) and amplitude (5.3 ± 0.6% versus 6.9 ± 0.4% for wild-type, *p* > 0.05) (Figure 3C–3H). At synapses, whereas Ca^2+^ transients were present in all wild-type animals, half of the *nca(lf)* mutants showed no Ca^2+^ transients at all (Figure 3B, *nca(lf)*, synapse, top trace). The rest of the *nca(lf)* mutants retained Ca^2+^ transient trains (Figure 3B, *nca(lf),* synapse, bottom trace). This resulted in an overall significant decrease of synaptic spike frequency (1.2 ± 0.7 spikes/min in *nca(lf)*) compared to wild-type synapses (2.6 ± 0.8 spikes/min, *p* = 0.029), and to the spike frequency of *nca(lf)* cell bodies (3.5 ± 0.6 spikes/min, *p* = 0.005). Remarkably, the remaining trains of Ca^2+^ transients in the *nca(lf)* mutants maintained temporally correlated in spike interval (5.2 ± 0.4 s) with the cell body of *nca(lf)* mutants (5.8 ± 0.2 s, *p* > 0.05). They also display comparable amplitude (6.8 ± 0.7%) to those in wild-type synapses (5.2 ± 0.7%, *p* > 0.05) (Figure 3C–3H). Thus the loss of NCA function disrupts the initiation of Ca^2+^ transients at synapses.

In *nca(gf)* mutants, HSN cell bodies also displayed trains of calcium spikes similar to those in wild-type animals in their frequency (3.3 ± 1.0 spikes/min, versus 3.2 ± 0.6 spikes/min for wild-type, *p* > 0.05), interval (5.3 ± 0.3 s, versus 8.6 ± 0.2 s for wild-type, *p* > 0.05) and amplitude (9.2 ± 1.4% versus 6.9 ± 0.4% for wild-type, *p* > 0.05) (Figure 3B–3E). At synapses, they all displayed trains of Ca^2+^ spikes that temporally correlated in frequency (2.7± 0.8 spikes/min versus 3.3 ± 1.0 spikes/min, *p* > 0.05), and interval (6.4 ±0.3 s for synapses versus 5.3 ± 0.3 s for cell bodies, *p* > 0.05) with those in *nca(gf)* cell bodies. However, the amplitude of Ca^2+^ transients was significantly increased at synapses (9.5 ± 1.3% for *nca(gf)* versus 5.2 ± 0.7% for wild-type, *p* = 0.029). Although the mean amplitude appears only moderately bigger than in wild-type animals, *nca(gf)* mutants exhibited a fraction of unusually large Ca^2+^ transients at synapses that were well above the range seen in wild-type animals (Figure 3H, red box).

In summary, both *nca(lf)* and *(gf)* mutants specifically altered Ca^2+^ transients at the presynaptic regions, indicating that under our assay conditions, NCA activity does not alter the excitation at HSN soma but affects presynaptic activity. The decrease of Ca^2+^ transients in *nca(lf)* suggests that NCA is required to initiate presynaptic activation in response to depolarization signals. The elevated Ca^2+^ transients in *nca(gf)* mutants further suggests that the gain-of-function mutations enhance NCA's activity in presynaptic activation.

### NCA Activity Depends on UNC-79 and UNC-80, a Large, Novel Protein

To identify proteins that modulate NCA activities, we performed a genetic suppressor screen for mutations that reverted locomotion defects of *nca(gf)* mutants (see Materials and Methods). We identified two extragenic suppressors that reverted *nca(gf)* coilers to fainters and fully suppressed their synaptic morphology defects (Figure S1). One suppressor, *hp424*, corresponds to *unc-79*, a gene encoding a large, novel protein [24]. Another suppressor, *hp369*, failed to complement *unc-80*, an uncloned mutant previously isolated by its locomotion phenotype [32] and later shown to confer hypersensitivity to halothane [30]. *unc-80* (*hp369*), as well as two previously identified *unc-80* alleles, *e1272* and *e1069*, exhibit recessive and fully penetrant fainter phenotypes identical to that of the *nca(lf)* double mutant (Video S6). We found that *nca(lf);unc-80* triple mutants are indistinguishable from either *nca(lf)* double mutants or *unc-80* single mutants in behavior (Video S7). Furthermore, all *nca(gf);unc-80* double mutants display the same fainter phenotype as *unc-80* single mutants (Video S8). Therefore NCA and UNC-80 function in the same genetic pathway, with *unc-80* mutations epistatic to *nca(lf)* alleles, suggesting that NCA activity depends on UNC-80.

*unc-80* was recently cloned based on the observation that RNAi knockdown of an open reading frame *F25C8.3* in wild-type animals resulted in a fainter phenotype and the identification of missense mutations in *F25C8.3* from *unc-80* alleles [36]. We confirmed that genomic fragments containing only *F25C8.3* rescued the fainter phenotype of *unc-80* mutants (Video S9) and reverted the *unc-80;nca(gf)* mutants from fainters to *nca(gf)* locomotion patterns (Video S10). Nonsense or splice junction mutations, which are all predicted to result in the loss of the protein function, were identified in three *unc-80* alleles (Figure S4A), confirming that *unc-80* corresponds to *F25C8.3*. The *unc-80* gene is predicted to encode multiple isoforms of a large protein that contain no known protein motifs. Uncharacterized UNC-80 homologues are present in *Drosophila*, mouse, rat, and human (Figure S4B), suggesting that UNC-80 is a member of a novel but conserved protein family.

We confirmed that *unc-80* also regulates calcium transients at synapses. The Ca^2+^ dynamics of *unc-80* mutants were essentially identical to those observed in *nca(lf)*. The HSN cell bodies displayed trains of Ca^2+^ transients with normal frequency (5.1 ± 1.3 spikes/min for *unc-80* versus 3.2 ± 0.6 spikes/min for wild-type, *p* > 0.05), interval (5.8 ± 0.2 s for *unc-80* versus 8.6 ± 0.2 s for wild-type, *p* > 0.05) and amplitude (7.7 ± 0.7% for *unc-80* versus 6.9 ± 0.4% for wild-type, *p* > 0.05) (Figure 3B–3E). Likewise, half of these animals showed silencing of Ca^2+^ transients at synapse regions (Figure 3B, *unc-80*, top trace), with an overall reduction in frequency (0.9 ± 0.7 spikes/min) when compared to *unc-80* cell bodies (5.1 ± 1.3 spike/min, *p* = 0.037), and to wild-type synapses (2.6 ± 0.8 spike/min, *p* = 0.032). The remaining trains of Ca^2+^ transients at synapses maintained temporally correlated with cell body transients in spike interval (5.6 ± 0.5 s for synapses versus 5.8 ± 0.2 s for cell bodies, *p* > 0.05). They were also comparable in amplitude with wild-type synaptic transients (4.9 ± 0.5% versus 5.2 ± 0.7% for wild-type, *p* > 0.05) (Figure 3B, *unc-80*, bottom trace, Figure 3C–3E). Therefore in addition to sharing behavioral phenotypes with *nca(lf)* mutants, *unc-80* mutants also displayed identical changes in presynaptic activation. This indicates that UNC-80 either mediates or functions together with the putative NCA channel to regulate presynaptic activation.

### NCA-1 and UNC-80 Are Expressed and Function in Neuronal Processes

**Figure 4:**
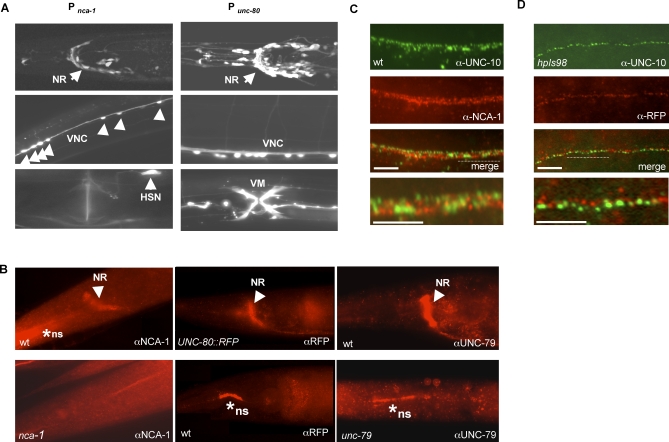
NCA-1, UNC-80, and UNC-79 Are Expressed in the Nervous System, Enriched at Nonsynaptic Regions Along Axons (A) A transcriptional GFP reporter driven by the *nca-1* promoter (left panels) or *unc-80* promoter (right panels) is active in neurons in the nerve ring (NR), and ventral nerve cord (VNC) motoneurons. Activity of the *nca-1* promoter is also seen in the HSN neuron whereas the *unc-80* promoter has activity in the vulval muscles (VM). (B) An antibody against NCA-1 showed specific staining (arrowheads) in the nerve ring (NR) and along nerve cords in wild-type (top left panel) animals that was absent in *nca-1(tm1851)* deletion mutants (bottom left panel). Similarly, anti-RFP antibody showed specific immunoreactivity in the nerve ring (NR) of UNC-80::RFP (*hpIs98*) expressing animal (top middle panel) but not in wild-type animals not carrying the transgene (bottom middle panel). * ns: nonspecific staining persisted in negative controls where animals do not express NCA-1 or UNC-80::RFP protein (out of the focal plane for the *nca-1* panel). An antibody against UNC-79 showed specific and similar staining at the nerve ring in wild-type animals (right top panel) that disappeared in *unc-79* mutants (right bottom panel). (C) Wild-type animals were co-stained with anti-NCA-1 antibody (red) and anti-UNC-10 (green). (D) *unc-80;hpIs98* animals co-stained with anti-RFP antibody (red) and anti-UNC-10 (green) showed poor colocalization. Scale bar: 5μm.

To determine how UNC-80 regulates the NCA activity, we first examined if they are both expressed or function in the same tissue. Green fluorescent protein (GFP) promoter reporter constructs, which contain their predicted upstream genomic sequences, revealed similar expression patterns in the nervous system, including many sensory neurons and all motoneurons, for both the *unc-80* and *nca-1* genes (Figure 4A). Expression of *nca-1* or *unc-80* by a pan-neural promoter (Text S1) was able to rescue the fainter phenotype of *nca(lf)* and *unc-80* mutants, respectively (Videos S11 and S12). Therefore, consistent with their expression patterns, both NCA-1 and UNC-80 are required in neurons. Furthermore, specific expression of NCA-1 by a GABAergic promoter *Punc-25* [37] rescued the active zone marker defects in GABAergic neurons of *nca(gf)* mutants (Figure S5), suggesting that NCA-1 functions cell-autonomously. Hence both NCA-1 and UNC-80 function in neurons.

NCA and UNC-80 may regulate presynaptic activation through either conducting Ca^2+^ transients at synapses, or transmitting depolarization signals along axons. To investigate these possibilities, we further examined their subcellular localization. With an NCA-1–specific antibody, we observed dense and punctate staining in the nerve ring, a synapse-rich region at the central nervous system of C. elegans, termed nerve ring, and along the ventral and dorsal nerve cords that are comprised of inter- and motoneuron processes in wild-type animals (Figure 4B, upper left panel). These staining signals disappeared completely in *nca-1(gk9)* deletion mutants (Figure 4B, lower left panel). The punctate staining pattern suggests a subcellular enrichment of NCA-1 protein along axons. We therefore examined the localization of NCA-1 relative to the presynaptic termini using antibodies against a vesicle protein, SNB-1; an active zone protein, UNC-10; and a presynaptic kinase, SAD-1. Along both the dorsal and ventral nerve cords, we observed mostly non-colocalizing staining patterns between NCA-1 and all presynaptic proteins (Figure 4C and Figure S6A), suggesting that NCA-1 is enriched at specific regions along motoneuron axons but not at synapses.

The subcellular localization of UNC-80 was examined using a functional P*unc-80-*UNC-80::RFP construct that rescued the fainter phenotype to the same degree as untagged genomic *unc-80* (Text S1 and unpublished data). *unc-80* mutants carrying *hpIs98*, an integrated transgenic array of P*unc-80-*UNC-80::mRFP, were stained with antibodies against RFP. We observed specific and punctate staining signals at the nerve ring and along the nerve processes (Figure 4B, wild-type as negative controls, Figure 4D) that do not colocalize with presynaptic proteins (Figure 4D and Figure S6B). This UNC-80::RFP staining pattern is highly reminiscent to that of NCA-1, suggesting that both NCA-1 and UNC-80 proteins are enriched at non-synaptic regions along nerve processes (Figure 4C and 4D). This expression pattern is most consistent with NCA-1 and UNC-80 functioning together to transduce depolarization signals from neuronal cell bodies.

### UNC-79, UNC-80, and NCA-1 Facilitate Each Other's Localization

**Figure 5:**
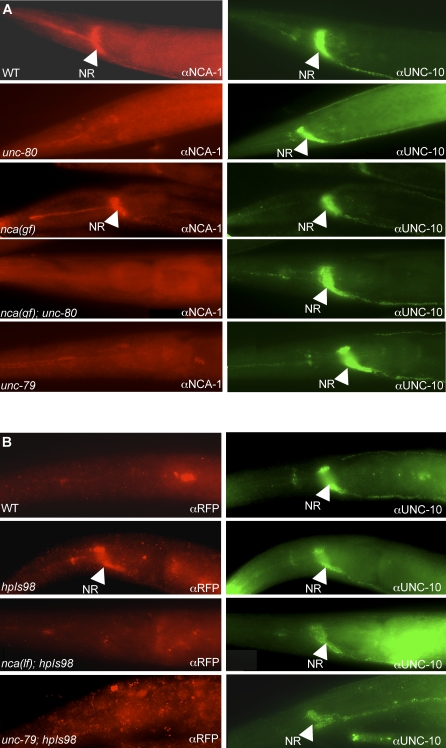
NCA-1, UNC-80, and UNC-79 Depend on Each Other for Localization (A) Wild-type (WT), *nca(gf)hp102*, *unc-80(e1272)*, *hp102;unc-80(e1272)*, and *unc-79(e1279)* animals co-stained with anti-NCA-1 (red) and anti-UNC-10 antibodies (green, as internal staining control). NCA-1 staining was present in wild-type and *hp102* animals but disappeared in *unc-80*, *unc-79*, and nca(gf)*hp102;unc-80* animals. (B**)** Staining with anti-RFP antibodies in wild-type (negative control), *hpIs98* (UNC-80::RFP), *nca(lf);hpIs98*, and *unc-79;hpIs98* animals (left panels). Specific nerve ring staining (arrow) of UNC-80::RFP disappeared in *nca(lf)* and *unc-79* animals. UNC-10 staining was present in the same animals (right panels). Scale bar: 5 μm.

To further determine how UNC-80 regulates NCA-1 activity, we examined the distribution of NCA-1 in *unc-80* mutants, and vice versa. NCA-1 staining was eliminated or greatly reduced in multiple *unc-80* alleles (Figure 5A, *unc-80* panel). *hpIs98* (P*unc-80*-UNC-80::RFP) restored NCA-1 expression at the nerve ring and along the nerve cords in *unc-80* mutants (Figure S7A), indicating that UNC-80 is both necessary and sufficient to localize NCA-1 along axons. While ample NCA-1 staining signals were present in *nca(gf)* mutants, the staining was also eliminated or greatly reduced in *nca(gf);unc-80* mutants (Figure 5A), suggesting that both wild-type and gain-of-function NCA-1 proteins depend on UNC-80 to localize along the nerve processes. *nca-1* transcripts were present at wild-type level in *unc-80* mutants (Figure S7B). Together with the fact that no obvious UNC-80::RFP signal was detected in neuronal cell bodies (unpublished data), these data indicate that UNC-80 regulates NCA-1 post-transcriptionally, perhaps through reduced translation of NCA-1 proteins or defective trafficking, clustering, or stabilization of NCA along axons. In *nca(lf)* mutants, UNC-80::RFP staining was also significantly reduced (Figure 5B, *nca(lf);hpIs98* panel), suggesting that UNC-80 localization along the axon is also dependent on the presence of NCA protein. This NCA-1–dependent localization of UNC-80::RFP, together with the fact that no transmembrane motifs are present in UNC-80, is consistent with the possibility that UNC-80 functions as an auxiliary subunit that regulates the transport, stability, or clustering of NCA at the membrane.

UNC-79 is another large protein with no known motif that has been implicated in the processes controlled by NCA-1, NCA-2, and UNC-80. *unc-79* loss-of-function mutants also have a fainter phenotype, and have been reported to contain lower than normal levels of NCA-1 protein by Western blot analyses [24]. As for *unc-80* mutants, we observed reduced or completely diminished NCA-1 staining in the *unc-79* mutants (Figure 5A, lower panels). Interestingly, UNC-80::RFP axonal staining was also absent in the *unc-79* mutants (Figure 5B, lower panels), suggesting that UNC-79 is another auxiliary protein that facilitates NCA-1 localization along the axon. We generated an antibody against the UNC-79 protein, and observed punctate staining in ventral cord and nerve ring processes, consistent with the possibility of coexpression with UNC-79 and NCA-1 (Figure 4B and Figure S8). When the same antibody was used to stain *nca(lf)*, *unc-80*, and *unc-80;nca(lf)* mutants, no UNC-79 staining was detectable in neuronal processes (Figure S9). Thus, NCA-1/2 and UNC-80 appear to also facilitate the localization of UNC-79 protein. These results are consistent with the possibility that UNC-79, like UNC-80, also functions as an accessory subunit or another regulatory interactor with the NCA channel.

### UNC-80 Enhances the Effect of NCA-1 in Transfected HEK293T Cells

**Figure 6:**
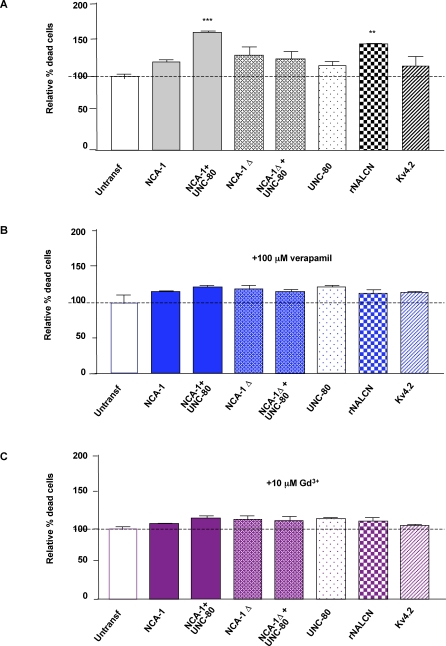
Co-Transfecting NCA-1 and UNC-80 Induces NALCN-Like Cell Death in HEK293T Cells The results of propidium iodide cell death assays in HEK293T cells are graphically represented. Assays were done on mock-transfected (untransf) HEK293T cells, or cells transfected with various combination of DNA constructs that express UNC-80, NCA-1, truncated NCA-1 (NCA-1Δ), the rat (rNALCN) homologue of NCA-1, and the Kv4.2 potassium channel (as an additional control), either in the absence of any blockers (A), or in the presence of 100 μM verapamil (B) or of 10 μM gadolinium (C). The graph presented resulted from three independent sets of experiments. Statistical significance was analyzed by one-way ANOVA followed by post-hoc Student-Newman-Keuls tests. **p < 0.01, significantly different from the mock-transfection control.

To further investigate whether NCA-1 and UNC-80 proteins might function together to promote NCA channel activity, we analyzed NCA function in a heterologous cell system. It was shown previously that the expression of mammalian NALCN induced constitutive cation leak currents when transfected in HEK293T cells [26]. These currents were attributed to the NALCN channel activity, because they were inhibited by verapamil or gadolinium, two blockers for the endogenous, NALCN-mediated Na^+^ leak currents in hippocampal neurons [26]. In our experiments, these currents appeared to induce cell death in the transfected cells, because significantly increased cell death was observed 48 h after HEK293T cells were transfected with cDNAs expressing NALCN (144.3 ± 1.8%, normalized against untransfected cells, *p* < 0.01). This effect was not induced by the expression of other channels (e.g., for Kv4.2, 113.7 ± 13.9%, *p* > 0.05), and was abolished when transfected cells were incubated with 100 μM verapamil or 10 μM gadolinium (Figure 6) (see Materials and Methods), suggesting that the cell death was indeed associated with the NALCN channel activity.

Using this same assay, we examined whether C. elegans NCA-1, alone or together with UNC-80, exhibited similar activities in HEK293T cells (Figure 6). cDNAs encoding the longest isoform for NCA-1 and UNC-80 were maintained in a low–copy number expression vector (Text S1). Transfecting with either the NCA-1 or UNC-80 expression construct alone did not cause an increase in the lethality of the host cells (NCA-1: 119.3 ± 4.1%, and UNC-80: 114.3 ± 6.7%, *p* > 0.05). In contrast, co-transfection of NCA-1 and UNC-80 constructs induced significant cell death (159.3 ± 2.9%, *p* < 0.01). This effect was abolished when the UNC-80 expression vector was co-transfected with a NCA-1 clone carrying a deletion in the coding region (122.7 ± 11.6%, *p* > 0.05). Moreover, the increased cell death in NCA-1 and UNC-80 co-transfected cells was also blocked in the presence of 100 mM verapamil (120.3 ± 3.3%, *p* > 0.05) or 10 mM gadolinium (112.7 ± 3.7%, *p* > 0.05). Therefore the co-expression of UNC-80 and NCA-1 induced the same effect, with similar blocker responses as NALCN in HEK293T cells, suggesting that the putative NCA/UNC-80 channel complex likely shares similar ion leak properties.

## Discussion

### A Putative NCA Channel Transmits Depolarization Signals in C. elegans Neurons

**Figure 7:**
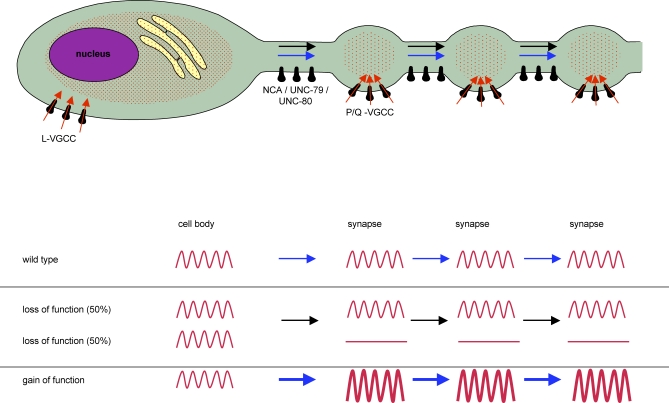
A Model of NCA-1/UNC-80 Function in Neurons Top: A schematic representation of a C. elegans neuron with *en passant* synapses. Ca^2+^ transients in the cell body are likely contributed by influx from L-type VGCCs and release from the intracellular calcium pools. At the synapse, depolarization signals (red waves) initiate calcium influx through P/Q-type VGCCs at the active zone. NCA channels (NCA-1, NCA-2, UNC-80, and UNC-79) regulate membrane excitability along the axon to allow the propagation of some depolarization signals. Bottom: In *nca(lf)* mutants some depolarization signals fail to propagate along the axon while others can still reach synapses. In *nca(gf)* mutants, the signals are amplified, resulting in increased synaptic activity, which indirectly regulates active zone distribution.

We have shown here that the NCA-1 and NCA-2 proteins are required redundantly for synaptic activity. Both the reduction of postsynaptic currents at GABAergic and cholinergic NMJs, and the decrease of Ca^2+^ transients at serotonergic NMJs in *nca(lf)* mutants suggest a decreased presynaptic activity in the absence of the putative NCA channels. The calcium imaging analyses further suggest that this synaptic defect is related to a failure to initiate presynaptic activity. In wild-type animals, the calcium spikes at HSN cell bodies and synapses are temporally correlated. In both *nca(lf)* and *unc-80* mutants, at least under our assay conditions, despite the normal calcium dynamics in cell bodies, the number of Ca^2+^ transients was reduced at synapses. The NCA channel is unlikely to conduct Ca^2+^ transients at synapses, since the remaining transients in *nca(lf)* mutants were normal in amplitude and maintained temporal correlation with the depolarization signals in cell bodies. Together with their nonsynaptic localization along nerve processes, these results strongly indicate that NCA channel activity is required to transmit depolarization signals to synapses (Figure 7).

Depolarization signals may propagate actively or spread passively along axons. Lacking typical voltage-gated sodium currents, the passive model, conceivable for neurons with short axons or axons with a high input resistance membrane property [38], was proposed for C. elegans sensory neurons [12]. Mouse NALCN mediates Na^+^ leak in hippocampal neurons [26]. A similar property for the NCA channel would allow it to drive the membrane potential close to its excitation threshold at specific regions along C. elegans neurites, facilitating the activation of other channels along axons or around synapses. This model is consistent with the presence of the active propagation of depolarization signals in C. elegans motoneurons.

Interestingly, the silencing of Ca^2+^ transients in *nca(lf)* and *unc-80* mutants is incomplete; however, the molecular lesions in these mutants predict severe loss of protein functions. All *nca(lf)* and *unc-80* alleles are behaviorally indistinguishable from each other and fully penetrant for the fainter phenotype, which strongly argues against an allelic effect on phenotype penetrance. The partial loss of Ca^2+^ transients and the variable degree of the decrease of mPSC frequency in *nca(lf)* mutants therefore more likely suggest that while some depolarization signals depend on NCA channels to induce presynaptic activation, other signals reach synapses independently of NCA activity (Figure 7).

It is worth noting that although we detected two distinct, active versus quiescent populations in *nca(lf)* and *unc-80* mutants in our physiological analyses, there is little behavioral variability among individual animals. Because every animal alternates between a state of normal sinusoidal movement and quiescence, we speculate that C. elegans neurons alternately fire NCA/UNC-80–dependent and -independent depolarization signals. Perhaps due to the necessary experimental manipulation (such as immobilization of the animal) and the short assay time, we measured neuronal activity fixed in one “mode,” resulting in the appearance of two distinct populations.

### The Effects of Gain-of-Function Mutations on Neuronal Excitability

In addition to the synaptic phenotypes observed in the loss-of-function mutants, we also observed behavioral and synaptic phenotypes in the *nca* gain-of-function mutants. Specifically, we found that these mutant animals showed a coiler uncoordinated phenotype, and exhibited larger calcium transients at synaptic sites. Gain-of-function mutations in NCA-1 do not affect the temporal correlation of calcium transients between HSN cell bodies and synapses. Whole-mount staining with antibodies against NCA-1 showed no obvious changes in the subcellular distribution or intensity of the staining signals in *nca(gf)* mutants, indicating that these mutations likely alter the activity rather than the abundance of the NCA-1 protein. The calcium imaging phenotype is consistent with the NCA(gf) channel further increasing the membrane excitability, which leads to enhanced activation of calcium channels at HSN synapses (Figure 7).

The *hp102* mutation alters a conserved amino acid flanking the IS6 transmembrane domain. This coincided with a hot spot region for identified gain-of-function alleles for several VGCCs. In several cases, these gain-of-function mutations lead to slowed inactivation, subsequently prolonging the duration of the corresponding currents [18,39,40]. If the *hp102* mutation leads to a further increase of the leak through the NCA channel, it could indeed bring the neuronal membrane to a hyper-excitable state. Expressing the mouse NALCN carrying the *hp102* equivalent mutation (NALCN(R329Q)) was able to induce similar locomotion defects as NCA-1(gf) proteins in C. elegans (Text S1 and Video S13), suggesting that *hp102* mutation may induce similar property changes in all NCA family channels.

### Both UNC-80 and UNC-79 Regulate the Putative NCA Channel through Localizing the Pore-Forming Subunit

Another gene with a loss-of-function fainter phenotype, *unc-80*, encodes a novel protein with a critical role in NCA channel function. Based on behavioral and physiological characterization of mutants, UNC-80 appears exclusively to be exclusively involved in NCA-mediated functions. Not only do *unc-80* mutants show identical phenotypes as *nca(lf)* mutants, they do not enhance *nca(lf)* mutants, and they suppress defects exhibited by *nca(gf)* mutant. By contrast, *unc-80* mutants do not phenocopy VGCC loss-of-function mutants or display obvious genetic epistasis with VGCC gain-of-function mutants (Table S1 and Video S14). These genetic interactions indicate that UNC-80 function is specifically required for NCA channels.

UNC-80 regulates NCA channel function at least in part by localizing the putative pore-forming NCA-1 subunit to the membrane. *nca-1* transcripts are present in normal levels in *unc-80* mutants, suggesting that UNC-80 regulates NCA-1 post-transcriptionally. The similar and interdependent subcellular localization pattern of NCA-1 and UNC-80 implies that UNC-80 is a likely subunit of the NCA channel to transport, anchor, or stabilize the pore-forming subunit NCA-1 along axons. With close homologues present in all animals, proteins in the UNC-80 family likely play a conserved role in regulating the localization of the NCA family channels.

We observed identical genetic interaction between *nca* and *unc-79* mutants, and identical interdependent localization of NCA-1 and UNC-79 proteins. *unc-79* encodes another large but evolutionarily conserved protein with no known protein motifs [24]. Similar to *unc-80*, loss-of-function mutations in the *unc-79* gene lead to not only the same fainter phenotype as *nca(lf)* mutants, but also a complete suppression of *nca(gf)* locomotion defects and the disappearance of NCA-1 and UNC-80 along nerve processes. Furthermore, UNC-79 is dependent on the presence of both NCA and UNC-80 for its localization along neurites. Therefore, both UNC-80 and UNC-79 are likely conserved auxiliary components of the NCA family channels.

### Are the Functions of NCA Channels Conserved in Mammalian Neurons?

NCA-1 and NCA-2 have close sequence homologues in other invertebrate and vertebrate, including human. The mammalian member of this family, NALCN, has recently been characterized physiologically in HEK293 cells [26]. In spite of its sequence homology and similar topology to the pore-forming α1 subunits of VGCCs, NALCN forms a voltage-insensitive and nonselective cation channel.

Two lines of indirect evidence support the hypothesis that C. elegans NCA and its mammalian homologues share common functional properties. First, C. elegans NCA proteins show at least similar properties to NALCN proteins when heterologously expressed in mammalian cell culture. In HEK293T cells, transfecting NALCN, or co-transfecting NCA-1 and UNC-80 induced cell death that was blocked by the NALCN blockers verapamil and gadolinium. Conversely, expressing mammalian NALCN proteins in C. elegans could substitute functionally for the NCA proteins. Specifically, wild-type C. elegans expressing a mouse cDNA that carries the *hp102* equivalent mutation (NALCN(R329Q)) in neurons exhibited a locomotion pattern with exaggerated body bends, reminiscent of the *nca(gf)* mutants (Text S1 and Video S13). Thus C. elegans NCA and its mammalian homologues can mediate similar physiological functions, consistent with the possibility that NCA family proteins share similar channel properties.

Given the conservation in the functional properties of NCA family members, it is reasonable to speculate that these channels may also carry out similar functions in neurons. Our current studies suggest a specific function for the NCA channel in transmitting and regulating excitability along C. elegans neuronal processes, but do not rule out the possibility that NCA also controls neuronal firing. Since our calcium imaging analysis was performed under conditions that stimulated the constitutive firing of HSNs, an altered firing ability could be masked by the hyperactivation of neurons. While the enriched localization of NCA-1 and UNC-80 at nonsynaptic regions along axons is consistent with the propagation role of the NCA channel, we do not exclude the possibility that the reduced synaptic transmission at GABAergic and cholinergic NMJs in *nca(lf)* mutants may result from a combination of deficits in the propagation of depolarization signals, neuronal firing, and even vesicle release. The mouse NALCN affects the resting potential and controls the excitability/firing rate of hippocampal neurons [26]; whether it is also involved in excitation propagation, however, is not examined. Therefore, it will be interesting to determine the subcellular localization of mouse NALCN channels and to examine whether they are also involved in such processes in mammalian neurons.

## Materials and Methods

### Strains.

All strains were cultured at 22 °C unless specified otherwise. *hp102* was originally identified in a genetic screen for *hpIs3* defective mutants [31] and was outcrossed eight times against wild-type N2. *unc-80(hp369)* and *unc-79(hp424)* were identified in a *hp102* suppressor screen and outcrossed three times against N2. *e625*, *e1069*, and *e1272* were identified through abnormal locomotion in previous C. elegans screens [32]. *gk9* and *gk5* were generated by the Gene Knockout Consortium and were outcrossed three times against N2. *tm1591* was generated by National Bioresource Project for the Nematode and was outcrossed once against N2.

### Identification, mapping, and cloning of *hp102* and *unc-77(e625).*

*hp102* mutants were identified from an active zone marker *hpIs3* screen [31]. Based on both of its abnormal active zone marker distribution and locomotion defects, *hp102* was rough mapped to Chromosome IV between E03H12 (1.40 cM) and D2096 (3.74 cM) by SNP mapping against CB4856. During the mapping, we noticed that *unc(uncoordinated)-77(e625)*, an uncloned, previously identified locomotion defective mutant [32] that was linked to a similar region on Chromosome IV, showed similar locomotion and active zone marker defects as *hp102* mutants (unpublished data). We determined that *e625* and *hp102* were alleles of the same gene due to the genetic interactions displayed by these two mutants: while *hp102/e625* heterozygous animals showed fully penetrant coiling locomotion as either homozygous mutants, *hp102/+* or *e625/+* heterozygous mutants showed only slightly more exaggerated body bends compared with wild-type animals. This conclusion was confirmed when we mapped both mutants to the same genetic locus, rescued both mutants with the same genetic fragments, and identified mutations in the same open reading frame (see below).

*hp102* and *e625* mutations were then further fine-mapped between B0273 (1.74 cM) and C49A9 (3.08 cM) based on the following data. From *dpy-13unc-77/CB4865*, three out of 20 Unc non Dpy animals had their recombination breakpoints between B0273 (1.74 cM) and F38A5 (3.21 cM), placing *unc-77* to the right of B0273 (1.74 cM). From *unc-77bli-6/CB4856* animals, three out of three Unc non Bli recombinants and two out of two Bli non Unc recombinants had their recombination breakpoints between C49A9 (3.08 cM) and F38A5 (3.21 cM), placing *unc-77* to the left of C49A9 (3.08 cM). From *unc-5unc-77/CB4856* heterozygous animals, two out of two *unc-77* non *unc-5* recombinants had their recombination breakpoints between C31H1 (2.56 cM) and C49A9 (3.08 cM), placing *unc-77* between C31H1 (2.56 cM) and C49A9 (3.08 cM). Cosmids and PCR fragments amplified from the genomic sequence covering this region (for details, see Molecular biology section below) were injected into *hp102;hpIs3* and *e625;hpIs3* animals. Only DNA fragments covering the C11D2.6 (*nca-1)* genomic region rescued the locomotion and *hpIs3* marker defects. We further confirmed that *unc-77* corresponds to *nca-1* by sequencing the entire predicted genomic regions (all exons and introns) of *hp102* and *e625* mutants and identifying a single missense mutation in the coding region of each mutant (Figure 1).

Both *hp102* and *e625* mutants harbor gain-of-function mutations for *nca-1* because they both behaved as semi-dominant mutations; *hp102/+* or *e625/+* heterozygous mutants showed more exaggerated body bends compared to wild-type animals, but much less severe than homozygous or *hp102/e625* heterozygous animals. They also behaved dominantly over *nca-2* loss-of-function mutations, as *hp102;nca-2(gk5)* and *e625;nca-2(gk5)* mutants displayed the same behavior as *hp102* and *e625* homozygous mutants. Furthermore, PCR fragments amplified from the *nca-1* genomic region from *hp102* when expressed in wild-type animals induced the same coiling locomotory defects as *hp102* mutants (Video S4). Lastly, overexpression of the wild-type copy of *nca-1* rescued phenotypes induced by a gain-of-function mutation likely by replacing the mutated NCA-1 protein from its putative channel complex.

### Identification, mapping, and cloning of *unc-80.*

*hp102;hpIs3* mutants were mutagenized by EMS, and F2 progenies displaying noncoiler locomotion patterns were recovered as candidate suppressors. Each candidate suppressor line was rescreened and confirmed by their rescuing of *hpIs3* marker defects. We backcrossed each suppressor line to wild-type animals: if coilers could be recovered from the F2 generation, the suppressor was considered as extragenic. If the coilers could not be recovered, the suppressor line was then crossed into the *nca-2(gk9)* background. If they showed fainter phenotype, the suppressor was confirmed as intragenic revertants. From the progenies of 13,000 mutagenized F1 hermaphrodites (equivalent to 26,000 mutagenized haploid genomes), we identified four intragenic suppressors reverting *hp102* mutants to wild-type locomotion. We also identified multiple alleles of two different extragenic suppressors that reverted *hp102* animals to fainters, and failed to complement *unc-80* and *unc-79* mutants, respectively**.**

*unc-80* was first rough mapped between F21D9 (21.82 cM) and F38A6 (27.08 cM) on Chromosome V through two-factor SNP mapping against CB4856. Two out of four *unc-80* recombinants from *unc-51unc-80*/CB4856 broke between Y113G7A (24.71 cM) and F38A6 (27.08 cM), placing *unc-80* to the right of Y113G7A (24.71 cM). 0/9 Rol non Unc recombinants from *unc-51rol-9/unc-80* animals segregated *unc-80*, placing it to the right of *rol-9* (25.124 cM, pKP5057). In two-factor mapping against *pha-4* and CB4856, we could not find breakage between *unc-80* and *pha-4* (25.60 cM), placing *unc-80* between 25.12 cM and 27.08 cM, tentatively near 25.60 cM. Clones that cover this region were generated by PCR to be tested for rescuing of *unc-80* mutants. Kim Schuske (University of Utah) determined that RNAi knockdown of *F25C8.3* (which lies within this region) in wild-type animals was able to induce a fainter phenotype (K. Schuske, personal communication). We generated and shared with the Schuske group DNA fragments spanning *F25C8.3* (see Text S1, Molecular biology; Trasngenic strains section) that rescued the fainter phenotypes in *unc-80* and *unc-80;hp102* mutants.

### Molecular biology; transgenic strains.

See Text S1.

### Immunocytochemistry.

Antibodies against aa1731–1914 of the predicted NCA-1d isoform, and a combination of aa506–608 and aa1205–1851 of UNC-79 were generated in rat (*Covance*). Whole-mount immunofluorescent staining was carried out as previously described [41]. Antibodies against NCA-1, UNC-79, mRFP (*Clontech*), SAD-1, SNB-1, and UNC-10 (M. Nonet, Washington University, St. Louis) were used in 1:10, 1:10, 1:200, 1:200, 1:100 and 1:2000 dilutions, respectively.

### Electrophysiology.

Dissections on young adult C. elegans were performed as described [14,42]. The integrity of the anterior ventral medial body muscle and the ventral nerve cord was visually examined, and muscle cells were then patched using fire-polished 4-MΩ resistant borosilicate pipettes (World Precision Instruments). They were clamped at −60 mV using an Axopatch 1D amplifier throughout experiments (Molecular Devices), and recorded using the whole-cell patch-clamp technique in previously described recording solutions [43] within 5 min following the dissection. Signals were filtered at 5 kHz, and digitized via a Digidata 1322A acquisition card (Molecular Devices). Data were acquired and analyzed using the pClamp software (Molecular Devices). After 10–60 s of recoding of spontaneous events, a highly resistant fire-polished electrode filled with 3 M KCl was brought close to the ventral nerve cord region anterior to the recorded muscle cell, and a 1-ms depolarizing current, generated by a S11B GRASS stimulator (Astromed) was applied to induce an evoked response. All recordings were performed between 5 and 10 min after the beginning of the dissection process. 

### Calcium imaging.

*Pcat-1-cameleon* was used to reveal relative Ca^2+^ concentrations in HSN cell bodies and synapses corresponding to those on vm2 muscles. Adults 24 h post L4 stage were immobilized by surgical glue on 2% agarose pads on microscope slides and covered with 1 ml of 10 mM HEPES (pH 7.1), a condition that stimulates constitutive egg-laying thus spontaneous activation of HSN neurons. Recording was carried out as previously described [35]. All recordings started within 2 min after animals were glued and lasted for 10 min. Data from HSN cell bodies and synapses were obtained simultaneously. Due to slight body movements during the recordings, some synapse datasets were incomplete and were not included in analysis. Spike detection, data analysis, and statistic analysis by Kolmogorov-Smirnov rank test (due to abnormal data distribution) were carried out as described previously ([44] and Text S1).

### HEK293T cell death assay.

HEK293T cells were grown in α-MEM (GIBCO) medium supplemented with 10% FBS (GIBCO) at 37 °C in a humidified atmosphere of 5% CO_2_, 95% air. Lipofectamine 2000 was used to transfect the HEK293T cells following the standard procedure (Invitrogen). 0.4 μg total DNA was used for each transfection experiment. Medium was replaced 4 h after transfection, during which the culture was split into three sets with equal density, two sets were exposed to 100 μM verapamil or 10 μM Gd^3+^, respectively.

Cell death assays were performed 48 h after transfection. Culture medium was replaced by extracellular solution containing 50 μg/ml of propidium iodide (PI) (Invitrogen). After 30 min incubation at 37 °C, fluorescence intensity in each well was measured with a plate reader (Victor3; PerkinElmer) as described previously [45,46]. The fraction of dead cells was normalized against the mock-transfected cultures.

## Supporting Information

Figure S1 *hp102* Mutants Affects Locomotion, Active Zone Marker Distribution at NMJs, and Egg-Laying Behavior, Which Were Fully Suppressed by *unc-80* Mutations (A) Images of the body morphology of *nca(gf)*, *nca(lf)*, *unc-80*, and *nca(gf);unc-80* mutants. The coiling position of *nca(gf)* was fully restored in *nca(gf);unc-80* mutants.
(B) Active zone marker morphology of DD GABAergic synapses in L2 larvae of wild-type, *hp102*, *unc-80*, and *nca-1(hp102); unc-80* animals, visualized by SYD-2::GFP (*hpIs3*). Inserts are magnified views of regions marked by the dotted line. *nca(gf)* mutants showed abnormal clustering (arrowheads) and gaps between active zone marker puncta. This defect was fully suppressed in *nca(gf);unc-80* mutants.
(C) Quantification of the average number (*n* = 10 for each strain) for *hpIs3* (∼1/3 of dorsal cord region), where *nca(gf);unc-80* mutants showed wild-type level puncta.
(D) Morphology of HSN synapses visualized by an active zone marker *wyIs12* also showed abnormal clustering in *nca(gf) hp102* mutants (bottom panels) compared to wild-type animals (top panels). This phenotype was rescued in *nca(gf);unc-80* mutants.
(E and F) *nca(gf) hp102* mutants display constitutive egg-laying, resulting in fewer eggs (E, the total number of eggs) and younger eggs (F, % of eggs eight cells or younger) retained in uterus of *hp102* animals compared to wild-type animals. *n* = 15, the number of eggs was counted from animals 24-h post L4 larval stage. Error bar: SEM. Statistic comparisons were performed against the wild-type dataset using the Tukey-Kramer multiple comparison test. *p < 0.01, **p < 0.001, Scale bar: 5μm.
(805 KB PDF)

Figure S2 HSN Cell Bodies Autonomously Generated Calcium Spikes Independent of Presynaptic Input Under the Assay Condition (10 mM HEPES, pH 7.1) A manuscript describing this work has been submitted. We present here only one piece of supporting evidence. In *unc-13(e51)* mutants, where synaptic transmission is severely abolished, HSN cell bodies were capable of generating trains of calcium spikes. (A) Representative cameleon trace displayed by HSN cell bodies of *unc-13(e51)* mutants as in Figure 3. (B) Calcium spike frequencies of HSN cell bodies in wild-type (wt) and *unc-13(e51)* mutants showed no statistically difference by the Kolmogorov-Smirnov rank test (*p* > 0.1). The number at the bottom of each bar represented the number of animals examined.
(52 KB PDF)

Figure S3 Raw Traces of YFP and CFP Recorded in HSN Cell Bodies and Synapses The left panel, top two lines, show raw YFP and CFP traces for HSN cell body recordings shown in Figure 3. The *x-*axis represents the recording time in seconds, and the *y-*axis represents fluorescent intensity in arbitrary units of pixel intensity. The ratio between YFP and CFP fluorescent signals at each time point was plotted against the same *x-*axis, resulting in a third trace that represents the YFP/CFP ratio change. Calcium spikes display a characteristic asymmetric shape, with a fast, linear rising phase followed by a slower, exponential decaying phase (arrows); whereas peaks due to noise, or random fluorescent ratio fluctuations, typically show a symmetric shape, with linear rising and decaying phases (arrowheads). Reciprocal changes in YFP and CFP intensity were observed for many ratio peaks (examples were shown by dashed lines), though reciprocity was sometimes obscured by motion artifacts caused by movement of the neurons during egg-laying. Despite the fluctuations in the absolute YFP and CFP intensity levels over the recording period, the YFP/CFP ratio-metric trace revealed similar ratio changes for most calcium spikes in wild-type animals, suggesting that the calcium spike size is insensitive to fluorescent baseline changes and accurately reflects changes in calcium concentration. The right panel shows raw YFP and CFP traces for HSN synaptic trace shown in Figure 3.
(49 KB PDF)

Figure S4 UNC-80 Encodes a Highly Conserved Novel Protein **(**A) A schematic representation of the gene structure of *unc-80 (F25C8.3)* adapted from the Wormbase. The exons are shown as pink boxes. The genetic lesions of *unc-80* alleles (*e1069*, *e1272*, and *hp369*) are shown. (B) Protein structure and similarity of UNC-80 family members. *C.e.*: C. elegans; *D.m.: Drosophila melanogaster*; *R.n.: Rattus norvagicus*; *M.m.: Mus musculus*; *H.s*.: *Homo sapiens*.
(57 KB PDF)

Figure S5 NCA-1 Functions Cell Autonomously in Neurons to Regulate Active Zone Morphology Active zone morphology was examined using the SYD-2::GFP marker *hpIs3* in wild-type and *nca(gf)* respectively. In wild-type animals, SYD-2::GFP puncta are round and regularly spaced, whereas *nca(gf)* animals show regions lacking puncta, as well as clustering of puncta. Genomic DNAs containing the *nca-1* gene, or expression of NCA-1 from a GABAergic neuron-specific promoter, restored the *hpIs3* phenotype of *nca(gf)* to wild-type morphology. Inserts show a higher magnification of the region underlined by the dotted line. Scale bar: 5 μm.
(246 KB PDF)

Figure S6 NCA-1 and UNC-80 Are Enriched at Nonsynaptic Regions **(**A) Wild-type animals were co-stained with anti-NCA-1 antibody (red) and either anti-SNB-1 or anti-SAD-1 antibodies (green). (B) *unc-80; hpIs98* (UNC-80::RFP) animals were co-stained with anti-RFP antibody (red) and either anti-SNB-1 or anti-SAD-1 antibodies (green). Poor colocalization was observed in all cases. Scale bar = 5 μm.
(366 KB PDF)

Figure S7 UNC-80::RFP Restores Localization of NCA-1 in *unc-80* Mutants (A) UNC-80 regulates NCA-1 protein localization. Wild-type and *unc-80* animals were stained with anti-NCA-1 (red) and anti-UNC-10 (green) antibodies. Punctate NCA-1 staining pattern along the ventral and dorsal nerve cords was observed in wild-type but diminished in *unc-80* animals. UNC-10 staining pattern was similar between wild-type and *unc-80* mutant animals. The expression of UNC-80::RFP from its own promoter (*hpIs98*) restored the punctate expression pattern of NCA-1 along the nerve cords.
(B) *unc-80* mutations do not affect the transcript level of *nca-1*. Total RNA was isolated from mixed staged wild-type, *unc-80*(*e1069)* and *unc-80(e1272*) animals, and the transcript levels of *nca-1* were analyzed by RT-PCR (with a control reaction containing no template). *nca-1* transcript was present at similar levels in all strains. Scale bar: 5 μm.
(480 KB PDF)

Figure S8 Endogenous UNC-79 Is Expressed in the Nervous System (A) An antibody against UNC-79 was used to stain wild-type (top panel) and *unc-79* animals (bottom panel). Specific staining was observed in the nerve ring of wild-type but not in *unc-79* mutant animals. *ns: non-specific staining. (B) The dorsal (top panel) and ventral (bottom panel) nerve cords of wild-type animals stained with anti-UNC-79 antibody showed punctate staining pattern.
(201 KB PDF)

Figure S9 UNC-79 Localization Depends on the Presence of UNC-80 and NCA Wild-type, *nca(gf)*, *nca(lf)*, *unc-80*, *nca(lf);unc-80*, and *nca(gf);unc-80* animals were simultaneously stained with anti-UNC-79 antibody. UNC-79 signals were detected in the nerve ring (NR) of wild-type and *nca(gf)* animals but disappeared in other mutants.
(399 KB PDF)

Table S1 Summary of Phenotypes Displayed by *nca* and VGCC Single and Double Mutants *nca(lf)* or *unc-80* mutants in combination with *cca-1*, *egl-19* (*lf* and *gf*) and *unc-2* mutants display enhanced or additive locomotion defects than either single mutants. Similarly, *nca(gf)* mutants in *unc-2* and *egl-19*(*lf)* backgrounds lead to more severe or additive locomotion phenotypes. In particular, *egl-19(gf)* mutations do not suppress or improve the locomotion defects of *unc-80* and *nca(lf)* mutants. *nca(gf)* mutations do not improve the locomotion defects of *egl-19(lf)* or *unc-2(lf)* mutants. This suggests that NCA/UNC-80 and VGCCs do not function in a linear pathway. Their genetic interactions are most consistent with these two types of channels regulating different aspects of neuronal excitation.
(37 KB DOC)

Text S1 Supporting Materials and Methods (53 KB DOC)

Video S1 The Locomotion Pattern of Wild-Type C. elegans
A wild-type animal displays sinusoidal forward and backward locomotion patterns, also called body bends
(797 KB MOV)

Video S2 The Locomotion Pattern of *nca(lf)* Mutants An *nca-2(gk5);nca-1(gk9)* mutant animal displays a locomotion deficit termed as “Fainter”. The animal was capable of moving in a sinusoidal fashion upon stimulation (by probing, shown in this video; or to survive, e.g., lack of food in their habitat, not shown), but stopped after only a couple of body bends.
(980 KB MOV)

Video S3 The Locomotion Pattern of *nca(gf)* Mutants *unc-77(hp102)*, *nca(gf)* animals display constitutive and exaggerated body bends that make them appear “coiling” during both forward and backward movements.
(835 KB MOV)

Video S4 *hp102* Encodes a Gain-of-Function Mutation in *nca-1*
A wild-type animal expressing *nca-1* genomic fragments harboring the *hp102* mutation displayed the exaggerated body bends similar to those by *unc-77(hp102)* mutants (Video S3).
(2.2 MB MOV)

Video S5 Calcium Imaging of an HSN Cell Body in a Wild-Type Animal 4× real time. The cameleon signal was pseudo-colored based on the YFP/CFP ratio value, with red representing the highest value. Each pink flash reflected the peak of one calcium spike.
(410 KB MOV)

Video S6 The Locomotion Pattern of *unc-80* Mutants An *unc-80(e1272)* mutant animal displayed the same fainter locomotion deficit as *nca-2(gk5);nca-1(gk9)* mutants (Video S2).
(981 KB MOV)

Video S7 *unc-80* Mutations Do Not Enhance the Phenotype of *nca(lf)* Mutants An *nca-2(gk5);nca-1(gk9);unc-80(e1272)* mutant animal displayed the same fainter locomotion deficit as in *nca(gk5);nca-1(gk9)* (Video S2) and in *unc-80(e1272)* (Video S6) mutants.
(985 KB MOV)

Video S8 *unc-80* Mutations Suppress the Phenotype of *nca(gf)* Mutants An *unc-77(hp102); unc-80(e1272)* mutant animal displayed the same fainter phenotype as in *unc-80(e1272)* mutants (Video S6).
(1 MB MOV)

Video S9 *F25C8.3* Rescues the Locomotion Deficit of *unc-80* Mutants An *unc-80(e1272)* mutant animal carrying an extrachromosomal array that consisted of genomic fragments covering the *unc-80* locus displayed continuous locomotion, and did not ‘faint'.
(807 KB MOV)

Video S10 *F25C8.3* Reverts the unc-80 Mutation-Mediated Suppression of *nca(gf)*
A *unc-77(hp102); unc-80(e1272)* mutant animal carrying an extrachromosomal array that consisted of genomic fragments covering the *unc-80* locus displayed continuous and exaggerated sinusoidal (coiling) locomotion pattern, similar to that of *unc-77(hp102)* mutants (Video S3).
(479 KB MOV)

Video S11 NCA-1 Functions in Neurons An *nca-2(gk5); nca-1(gk9)* mutant animal carrying an extrachromosomal array expressing NCA-1 Driven by a pan-neuronal promoter displayed continuous and sinusoidal locomotion, and did not ‘faint.'
(794 KB MOV)

Video S12 UNC-80 Functions in Neurons An *unc-80(e1272)* mutant animal carrying an extrachromosomal array expressing UNC-80 driven by a pan-neuronal promoter displayed continuous and sinusoidal locomotion, and did not ‘faint.'
(972 KB MOV)

Video S13 *hp102*-Equivalent Mutation in Mouse NALCN Functions as NCA(gf) in C. elegans
A wild-type animal carrying an extrachromosomal array that expresses mouse NALCN(R329Q) driven by a pan-neuronal promoter displayed exaggerated body bends, which is characteristic of *hp102* and *e625* animals.
(159 KB MOV)

Video S14 The Locomotion Pattern of *egl-19(ad695)* Gain-of-Function Mutants The mutant animals were hyperactive and egg-laying constitutive, but are behaviorally distinguishable from the “coiling” *unc-77(hp102)* mutants (Video S3).
(1 MB MOV)

## Abbreviations

VGCC: voltage-gated calcium channel
NMJ: neuromuscular junction
GFP: green fluorescent protein
