# Supplementary material for: A Putative Cation Channel, NCA-1, and a Novel Protein, UNC-80, Transmit Neuronal Activity in C. elegans
Source: PLoS Biol. 2008 Mar 11;6(3):e55. doi: 10.1371/journal.pbio.0060055 (PMC2265767; doi:10.1371/journal.pbio.0060055)
Supplement: Supplementary file 1 [file pbio.0060055.sg001.pdf]

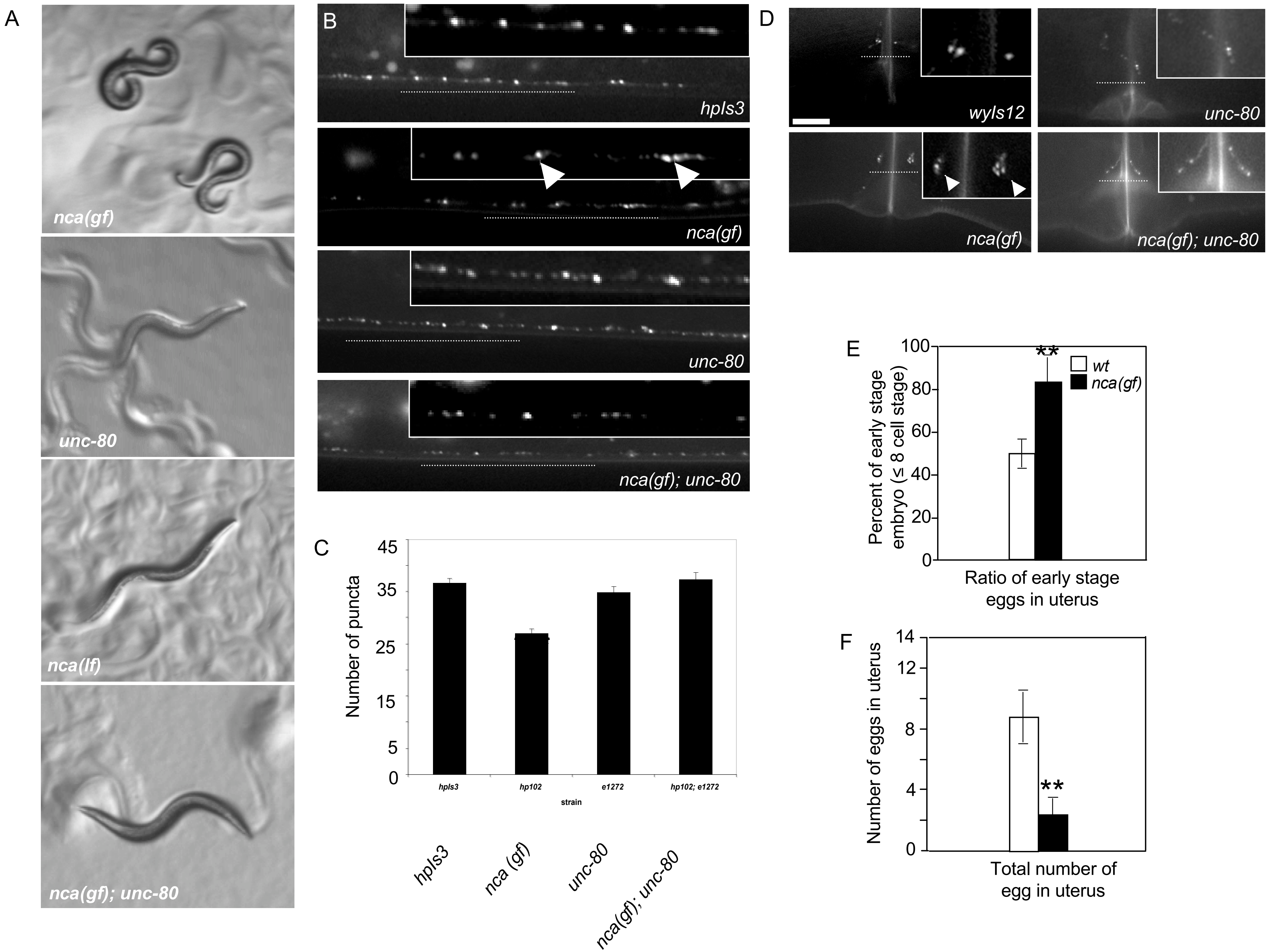
A
nca(gf)
unc-80
nca(lf)
nca(gf); unc-80
B
hpIs3
nca(gf)
unc-80
nca(gf); unc-80
C
Number of puncta
45
35
25
15
5
0
hpIs3
hp102
e1272
hp102; e1272
strain
hpIs3
nca (gf)
unc-80
nca(gf); unc-80
D
wyIs12
unc-80
nca(gf)
nca(gf); unc-80
E
Percent of early stage embryo (≤ 8 cell stage)
100
80
60
40
20
0
**
wt
nca(gf)
Ratio of early stage eggs in uterus
F
Number of eggs in uterus
14
12
8
4
0
**
Total number of egg in uterus
