# Supplementary material for: A Putative Cation Channel, NCA-1, and a Novel Protein, UNC-80, Transmit Neuronal Activity in C. elegans
Source: PLoS Biol. 2008 Mar 11;6(3):e55. doi: 10.1371/journal.pbio.0060055 (PMC2265767; doi:10.1371/journal.pbio.0060055)
Supplement: Supplementary file 3 [file pbio.0060055.sg003.pdf]

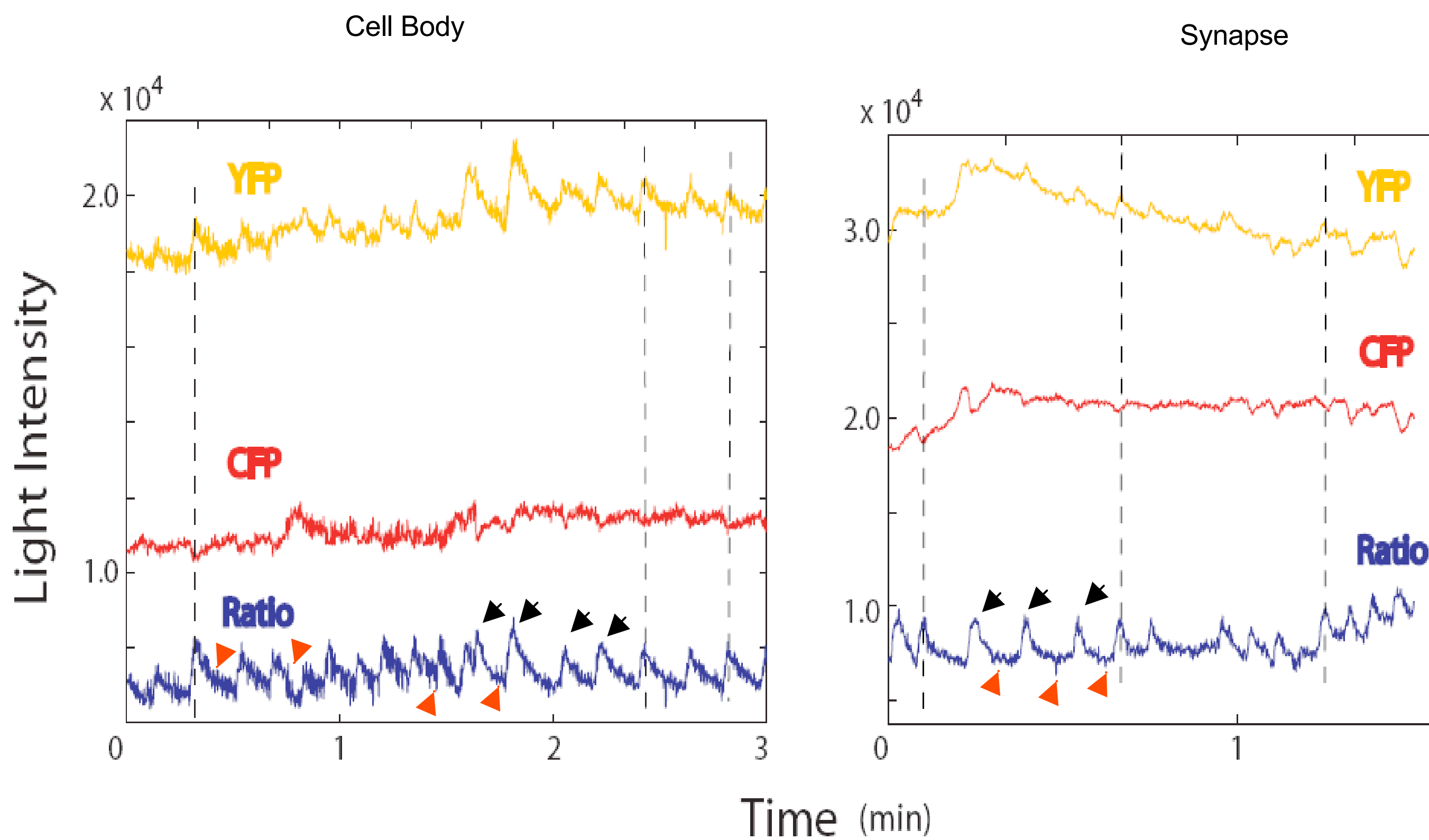

Cell Body
Synapse
x 10^4
2.0
1.0
YFP
CFP
Ratio
0
1
2
3
x 10^4
3.0
2.0
1.0
YFP
CFP
Ratio
0
1
2
Light Intensity
Time (min)
