# Supplementary material for: A Putative Cation Channel, NCA-1, and a Novel Protein, UNC-80, Transmit Neuronal Activity in C. elegans
Source: PLoS Biol. 2008 Mar 11;6(3):e55. doi: 10.1371/journal.pbio.0060055 (PMC2265767; doi:10.1371/journal.pbio.0060055)
Supplement: Supplementary file 4 [file pbio.0060055.sg004.pdf]

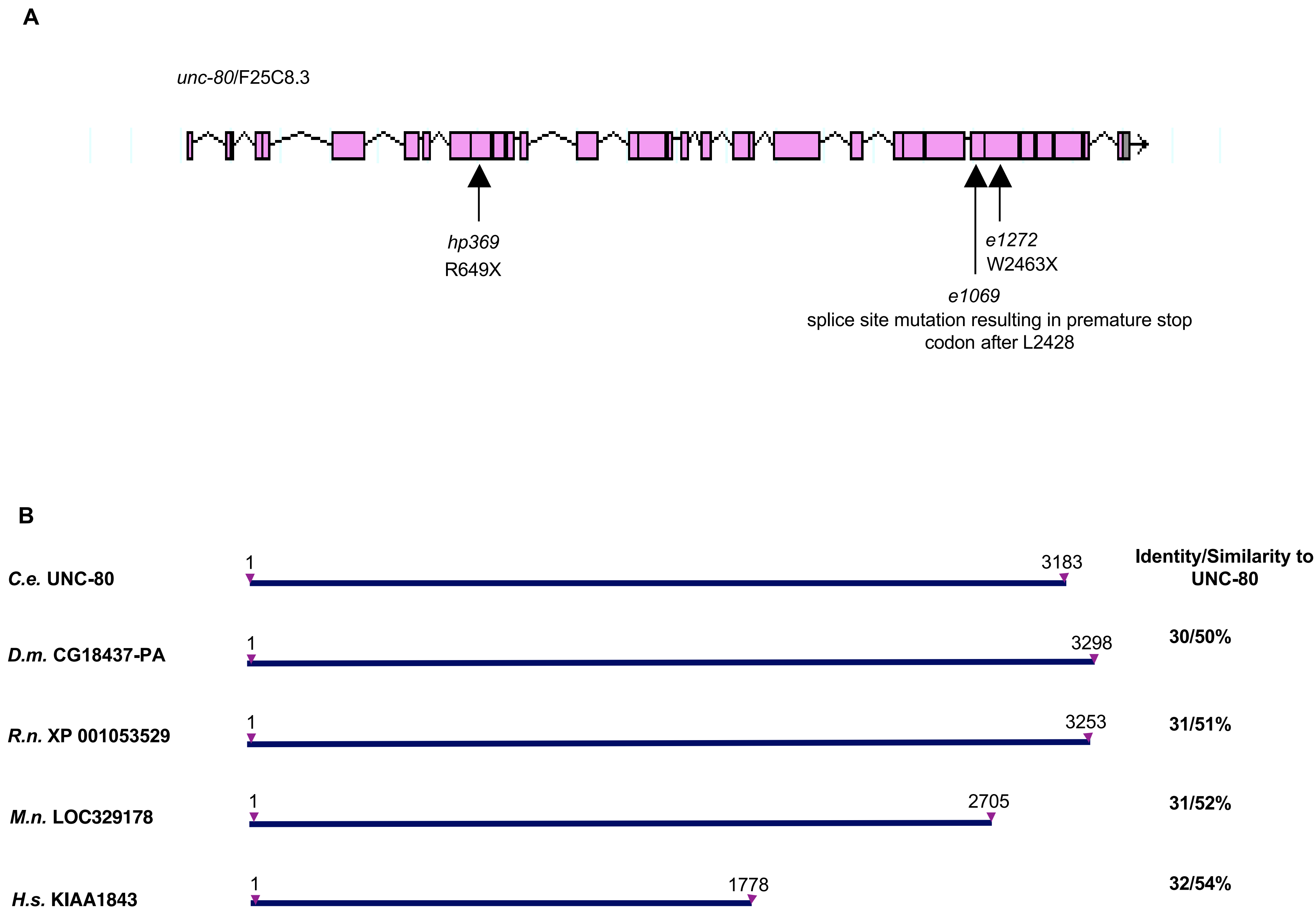
A
unc-80/F25C8.3
hp369
R649X
e1272
W2463X
e1069
splice site mutation resulting in premature stop codon after L2428
B
Identity/Similarity to UNC-80
C.e. UNC-80
1
3183
D.m. CG18437-PA
1
3298
30/50%
R.n. XP 001053529
1
3253
31/51%
M.n. LOC329178
1
2705
31/52%
H.s. KIAA1843
1
1778
32/54%
