# Supplementary material for: A Putative Cation Channel, NCA-1, and a Novel Protein, UNC-80, Transmit Neuronal Activity in C. elegans
Source: PLoS Biol. 2008 Mar 11;6(3):e55. doi: 10.1371/journal.pbio.0060055 (PMC2265767; doi:10.1371/journal.pbio.0060055)
Supplement: Supplementary file 5 [file pbio.0060055.sg005.pdf]

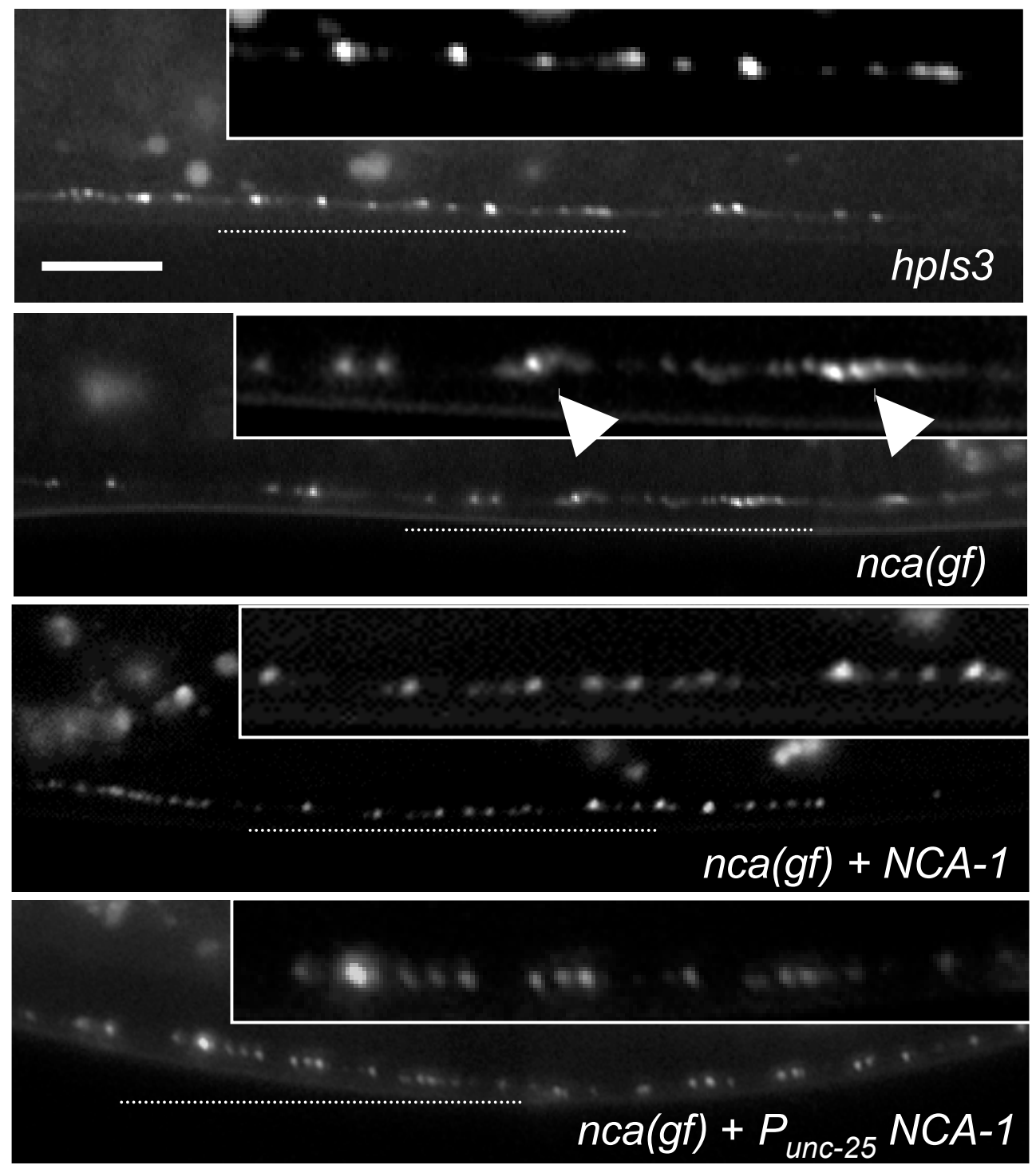

hpIs3
nca(gf)
nca(gf) + NCA-1
nca(gf) + $P_{unc-25}$ NCA-1
