# Supplementary material for: A Putative Cation Channel, NCA-1, and a Novel Protein, UNC-80, Transmit Neuronal Activity in C. elegans
Source: PLoS Biol. 2008 Mar 11;6(3):e55. doi: 10.1371/journal.pbio.0060055 (PMC2265767; doi:10.1371/journal.pbio.0060055)
Supplement: Supplementary file 6 [file pbio.0060055.sg006.pdf]

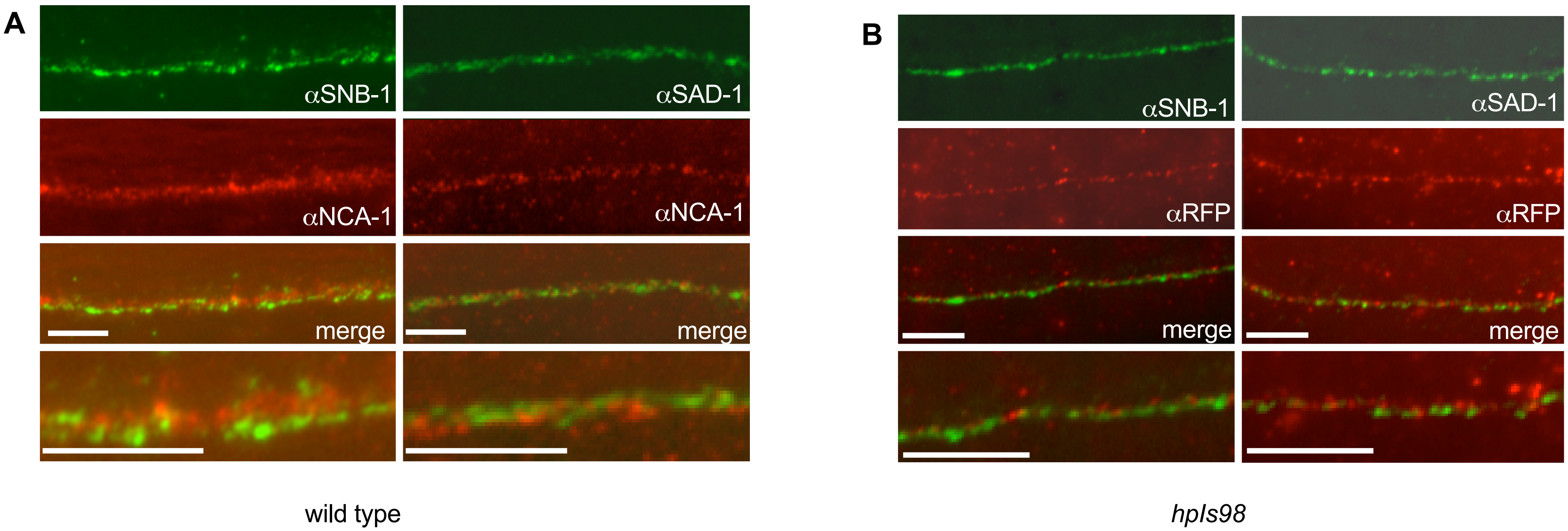
A
αSNB-1
αSAD-1
αNCA-1
αNCA-1
merge
merge
B
αSNB-1
αSAD-1
αRFP
αRFP
merge
merge
wild type
hpIs98
