# Supplementary material for: A Putative Cation Channel, NCA-1, and a Novel Protein, UNC-80, Transmit Neuronal Activity in C. elegans
Source: PLoS Biol. 2008 Mar 11;6(3):e55. doi: 10.1371/journal.pbio.0060055 (PMC2265767; doi:10.1371/journal.pbio.0060055)
Supplement: Supplementary file 7 [file pbio.0060055.sg007.pdf]

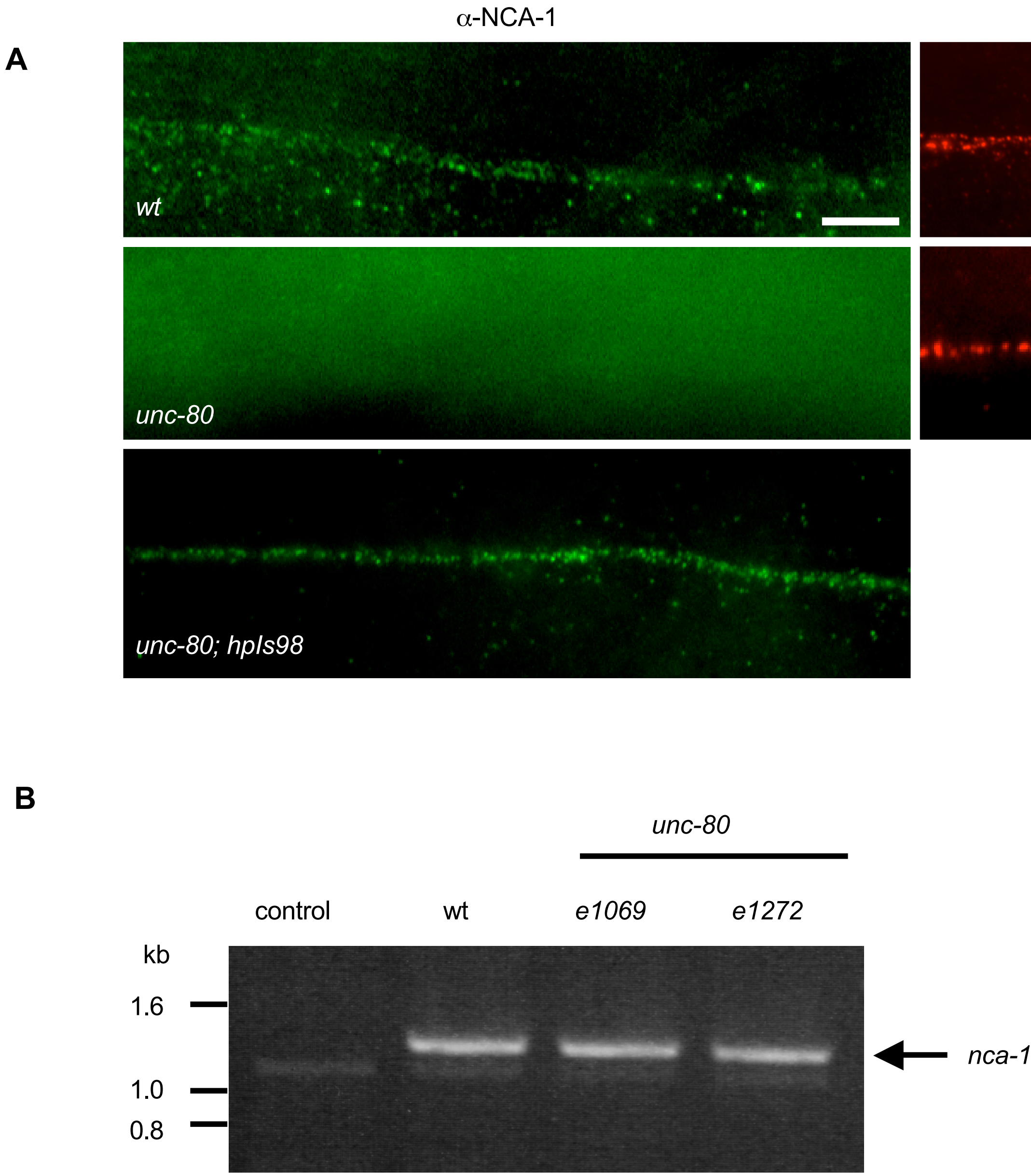
A
α-NCA-1
α-UNC-10
wt
unc-80
unc-80; hpIs98
B
unc-80
control
wt
e1069
e1272
kb
1.6
1.0
0.8
nca-1
