# Supplementary material for: A Putative Cation Channel, NCA-1, and a Novel Protein, UNC-80, Transmit Neuronal Activity in C. elegans
Source: PLoS Biol. 2008 Mar 11;6(3):e55. doi: 10.1371/journal.pbio.0060055 (PMC2265767; doi:10.1371/journal.pbio.0060055)
Supplement: Supplementary file 8 [file pbio.0060055.sg008.pdf]

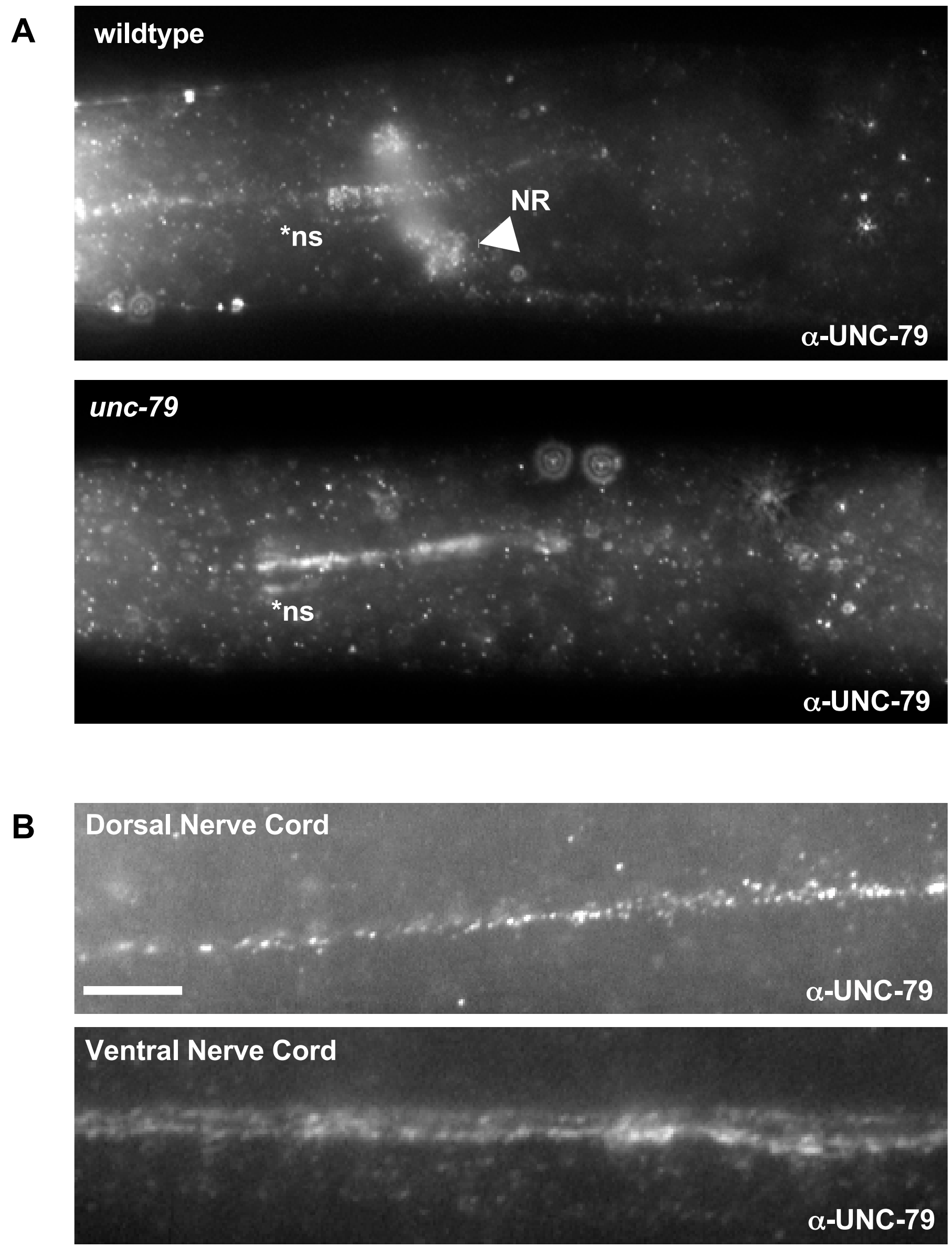

A
wildtype
NR
*ns
α-UNC-79
unc-79
*ns
α-UNC-79
B
Dorsal Nerve Cord
α-UNC-79
Ventral Nerve Cord
α-UNC-79
