# Supplementary material for: A Putative Cation Channel, NCA-1, and a Novel Protein, UNC-80, Transmit Neuronal Activity in C. elegans
Source: PLoS Biol. 2008 Mar 11;6(3):e55. doi: 10.1371/journal.pbio.0060055 (PMC2265767; doi:10.1371/journal.pbio.0060055)
Supplement: Supplementary file 9 [file pbio.0060055.sg009.pdf]

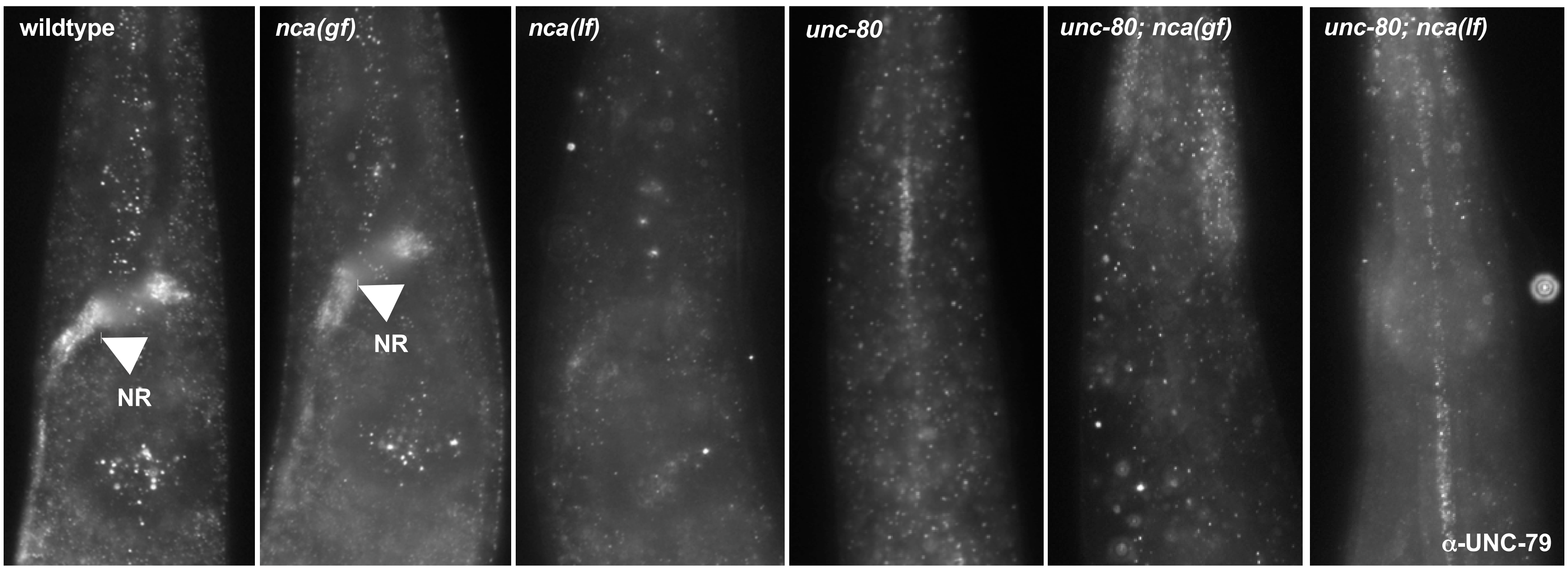
wildtype
NR
nca(gf)
NR
nca(lf)
unc-80
unc-80; nca(gf)
unc-80; nca(lf)
α-UNC-79
