# Supplementary material for: A Putative Cation Channel, NCA-1, and a Novel Protein, UNC-80, Transmit Neuronal Activity in C. elegans
Source: PLoS Biol. 2008 Mar 11;6(3):e55. doi: 10.1371/journal.pbio.0060055 (PMC2265767; doi:10.1371/journal.pbio.0060055)
Supplement: Supplementary file 10 [file pbio.0060055.sd001.doc]

**Supplemental text**

**Methods:**

**Molecular biology**

The promoter reporter construct for *nca-1,* pJH859, was generated by inserting 6.3kb of upstream genomic sequence that includes the ATG start codon plus the first 8 amino acids of the predicted C11D2.6a isoform into the SpeI/BamHI sites of pPD95.79 Fire vector. A single nucleotide deletion (T5044) at the predicted ATG start codon for the C11D2.6d isoform was created in this clone to prevent early translation, which is essential to allow the strong GFP reporter expression. The promoter reporter ‘constructs’ for *unc-80*, pJH880 and pJH881, consist of PCR fragments containing 3.6kb of genomic sequence immediately upstream of and including the ATG start codon of the predicted *F25C8.3a* open reading frame, fused in frame with GFP and followed by universal *unc-54* 3’-UTR sequences. The GFP and *unc-54* 3’-UTR sequences for pJH880 and pJH881 were generated from pPD95.69 and pPD95.77 vectors, respectively.

The rescuing ‘construct’ for *unc-77/nca-1* was a mixture of two DNA fragments, pJH986 and pJH901, PCR-amplified against *C. elegans* genomic sequences with primer pairs OZM390 (5’GCGAACTAGTGGATCGGCGAATTATCAAGTTGC3’)/OZM391 (5’ GCGAACTAGTCCTCCGACATCTCGAACAACGG3’) and OZM392 (5’GCGAACT AGTGCGTCGCCGATTCTCGAAGAC3’)/OZM393 (5’GCGAACTAGTCGTGTTTGT TACGACGCTCCGC3’), respectively. pJH986 contains 6.3kb of genomic sequence immediately upstream of the ATG start codon shared by the predicted C11D2.6a, b and c isoforms, followed by 7.7kb of N-terminal genomic sequence of the predicted C11D2.6 open reading frames. pJH901 contains 5.8kb of C-terminal genomic sequences of the predicted C11D2.6 open reading frames and 472bp sequences immediately downstream of the shared predicted TAG stop codon of C11D2.6c and d isoforms. These two fragments overlap by 4.0kb. The same primer sets were used to amplify DNA fragments using *hp102* genomic DNA as templates, sequencing confirmed for the *hp102* mutation, and co-injected into wild-type (N2) animals for dominancy tests.

Pan-neuronal expression of NCA-1 was achieved through co-injection of a plasmid pJH975 and the PCR fragment pJH901. pJH975 was generated by inserting 8.99kb of the N-terminal portion of *nca-1* genomic sequence (starting from the predicted ATG start codon of *C11D2.6d* isoform, PCR-amplified by an oligo pair OZM826 (5’GGGGATCCACATGT CGGAACGAAAAAAGAG3’)/OZM391 (5’GCGAACTAGTCCTCCGACATCTCGAA CAACGG3’) against *C. elegans* genomic DNA, into the BamHI/KpnI sites of pJH625, a pan-neuronal expression vector driven by P*F25B3.3* [1]. Expression of UNC-77/NCA-1 specifically in GABAergic motoneurons was achieved by co-injecting a DNA mix of pJH971 (which contains the same 8.99kb insert as pJH975 subcloned into the BamHI/KpnI sites of pJH387 (a GABAergic-specific expression vector driven by P*unc-25*) and the PCR fragment pJH901.

The rescuing ‘construct’ for *unc-80* was a mixture of three overlapping PCR-amplified *C. elegans* genomic fragments (pJH983, pJH984 and pJH985). pJH983, amplified with an oligo pair OZM967 (5’CCTGTCGACGTAGGTGACTTTCCGAGCTGCCA3’)/OZM973 (5’AGAGCTGATAGTCCCACTCTTCGAAACGGA3’), contains 3.6kb sequence immediately upstream of the predicted ATG start codon shared by F25C8.3a and b isoforms followed by 6.8kb of N-terminal genomic sequence for all predicted F25C8.3 open reading frames. pJH984, amplified with an oligo pair OZM594 (5’AAGGGCCCAATGCATGTT CGATACTTACAGCAC3’)/OZM679 (5’CGTGGTGAATCTTCCTCTGTGGCAG3’), contains 9.8kb of genomic sequence starting from the predicted ATG start codon of *F25C8.3* open reading frames. pJH985, amplified by an oligo pair OZM554 (5’TCCGTTTCGAAG AGTGGGACTATCAGCTCT3’)/OZM555 (5’GTTCGAATAGAATTGGTGGCCGTGT TTACCC3’), contains 12.1kb of the C-terminal portion of the predicted *F25C8.3* genomic sequence followed by 2.7kb of downstream sequence. Fragments pJH983-pJH984, as well as pJH984-pJH985, overlap by 3kb in genomic sequence. All three fragments were co-injected into *unc-80(e1272)* or *unc-77(hp102)*;*unc-80* mutants for rescuing experiments. The rescuing RFP-tagged *unc-80* genomic ‘construct’ is a mixture of pJH983, pJH984 and pJH916. pJH916 is a PCR-generated DNA fragment containing the same last 12.1kb C-terminal genomic sequence as pJH985 with mRFP (cherry) sequence inserted immediately before the stop codon, followed by *unc-54* 3’-UTR sequence. This fragment was generated based on a PCR-based tagging method [2]. Pan-neuronal promoter driven UNC-80 was achieved through co-injection of pJH994, a plasmid with the same insert as pJH982 subcloned into the pan-neural expression vector pJH625, together with pJH984 and pJH985.

The protein expression construct for antibody generation against NCA-1/UNC-77 is pJH997. The C-terminal region of the predicted C11D2.6d isoform was PCR-amplified from a cDNA clone, yk1314e03, using the primer pair OZM1059 (5’GGGGATCCGGACATTCT TCAATTTCACATGAGG3’)/OZM 758 (5’CCAAGCTTCTAATCAACAAGGGAATT CCACCA3’) and subcloned as a BamHI/HindIII fragment into the pMALC2X expression vector to generate pJH997. Constructs for the expression of UNC-79 recombinant proteins for antibody production are pJH523 and pJH690. pJH523 was generated by PCR-amplifying against cosmid E03A3 with primers OZM480 (5’ AaaggatccGCTGTATTCATATCA

AGTCTATGTGG 3’) and OZM481 (5’ AaagtcgacttaATAATCATCTCCACCAATTC TTCCAG 3’), and subcloned into the BamHI and SalI sites of pMal-c2x. pJH690 was generated by PCR-amplifying against *yk1042g09* with primers OZM523 (5’ AAAGGATCCTCAGGAGGTTACGGTAGACTCCGTG 3’) and OZM524 (5’ CCCGTCGACTTATTCCGCCAACTTAACCTTCAGATCACCAC 3’), and subcloned into the BamHI-HindIII sites of pMal-c2x. YC2.12 [3] was placed under the P*cat-1* promoter to generate the cameleon construct used to measure activity in HSN neurons.

For heterologous expression experiments in HEK293T cells, yk1314e03 (NCA-1 cDNA) and yk1260h06 (UNC-80 cDNA) cDNA clones in pME18S-FL vector were used (generated and provided by Drs Kohara, Suzuki and Sugano, Institute of Medical Sciences, University of Tokyo). These clones express the cDNAs from the SRα promoter. An expression vector of a deleted NCA-1 cDNA (pJH1161) was created by removing the ClaI fragment from yk1314e03, which removes aa847-1610 of NCA-1. The rat NALCN cDNA used for the HEK cell expression experiment was cloned in pTracer-CMV2 vector and provided to us by Dr. Ren (University of Pennsylvania).

The construct for expressing the mouse NALCN cDNA carrying the *hp102* mutation at the equivalent amino acid position under pan-neural promoter is pJH1160. 3 overlapping mouse NALCN partial cDNA fragments were amplified by RT-PCR, cloned into the T-vector and sequence verified. R329Q mutation was introduced into the corresponding cDNA fragment and the three pieces were sequentially subcloned into the BamHI site of the *C. elegans* expression vector pJH625. The complete insert for pJH1160 was re-sequenced to confirm the presence of the single mutation in the open reading frame.

**RT-PCR:**

Total RNA was isolated from mixed staged wild-type, *unc-80(e1272)* and *unc-80(hp369)* animals using a Trizol Kit (Invitrogen). First strand synthesis was performed using MMTV reverse transcriptase and 1μg of total RNA from each strain. The PCR reaction to detect all predicted *nca-1* transcripts was performed with oligos OZM684 (5’GGATC TGGCGATTCCTGATAC3’)/OZM685 (5’GTGATGAAGATTCGGCACCGA3’) which amplify a 1084bp region spanning exons 8 and 9.

**Transgenic lines:**

Extrachromosomal arrays (*hpEx* lines) were generated by co-injecting various DNA constructs with injection markers. The following are the strains and genotypes for the *hpEx* lines generated in this study: promoter reporter lines for the *nca-1*- ZM2154-2158 (*lin-15(n765);hpEx528-532[pJH859+LIN-15]*); promoter reporter lines for *unc-80* - ZM2159-2161 (*lin-15(n765);hpEx533-535[pJH880+LIN-15]*) and ZM2162-2164 (*lin-15(n765);hpEx536-538[pJH881+LIN-15]*); rescuing lines for *nca-1/unc-77(hp102)* were ZM1115,1116 (*nca-1/unc-77(hp102);juIs1; hpEx290,291[pJH986+pJH901+Pttx-3-GFP]*); rescuing lines for *nca-1(gk9);nca-2(gk5)* mutants were ZM2393 (*nca-1(gk9);nca-2(gk5); hpEx717[pJH986+pJH901+ Podr-1-GFP]*); rescuing lines (3 out of 25 lines) for panneural-expression of *nca-1* were ZM2429-2431 (*nca-1(gk9);nca-2(gk5);hpEx738-740[pJH975+pJH901+Podr-1::GFP]*); rescuing lines for *Punc-25*-driven NCA-1 were ZM2938-2985 (*nca-1(hp102);hpIs3;hpEx1047-1049[pJH971+pJH901+pRF4])*. Wild-type animals expressing *nca(gf)* genomic fragments were transgenic line ZM1658(*hpEx408[PCR genomic fragment of nca-1(hp102)+ Podr-1-GFP])*. Wild-type animals expressing Vgcnl1(gf) in neurons were transgenic lines, ZM3094 and 3095 (*hpEx1099-1100[pJH1160+Podr-1-GFP]*.

Rescuing lines for *unc-80(e1272)* were ZM2394 (*unc-80(e1272); hpEx718[pJH983+pJH984+pJH985+ Podr-1-GFP]*), ZM2389 (*unc-80(e1272); hpEx630[pJH983+pJH984+pJH916+ Podr-1-GFP]*), ZM2432 (*unc-80(e1272) ;hpEx741[pJH994+pJH984+pJH985+Podr-1::GFP]*) and ZM2275-2276 (*unc-80(e1272) ;unc-77(hp102); hpEx617-696[pJH983+pJH984+pJH985+ Podr-1-GFP]*). *hpEx630* was integrated to generate a stable transgenic array: ZM2390 (*unc-80(e1272); hpIs98 [pJH983+pJH984+pJH916+Podr-1-GFP]*). ZM2390 was outcrossed against *unc-80(e1272)* mutants twice before crossing into other genetic backgrounds.

*unc-77(hp102)*, *nca-2(gk5); nca-1(gk9), unc-80(e1272)* and *unc-80(e1272);unc-77(hp102)* mutant animals carrying the cameleon marker expressed in HSN neurons were generated by crossing the various mutations into an integrated cameleon transgenic array *ljIs65[Pcat-1-iYC1.12]*.

**Calcium imaging analysis:**

General guidelines for image acquisition and data analysis were described previously [4]. Briefly, time-lapse image data were acquired using a 63X water lens and the Metavue program (Molecular Devices). The total intensity of light emitted from the two cameleon fluorophores was calculated by a JAVA program Jmalyze. Photo-bleaching derived baseline-decay was corrected by a MATLAB script, RA, and calcium spikes were identified and analyzed using a MATLAB script, SFS. All computer program-identified spikes were re-examined and confirmed individually and manually based on the total ratio of spike above baseline noise, and the asymmetric (fast rising and slow decay) shape of the spikes.

The total spike number divided by the total imaging time is referred to here as Spike Frequency (#/min). In most cases, calcium transients distribute as periodic trains of calcium spikes, with the time interval between two consecutive spikes within the train following a normal distribution curve by lillietest and jb tests. The Spike Interval presented here refers to the intervals between spikes within these periodic trains, therefore largely measures the periodicity of events within the trains. The Spike Size (ratio change) was calculated by dividing the difference of the yellow/cyan ratio between the high and low points, with that of the low point of a spike. Histograms refer to the mean and standard error of the mean (SEM); statistical significance was calculated using the non-parametric Kolmogorov-Smirnov rank test due to non-normal data distribution.

**Supplemental Movies for *C. elegans* of various genetic backgrounds and calcium imaging:**

**Video S1. The Locomotion Pattern of Wild-Type *C. elegans***

A wild-type animal displays sinusoidal forward and backward locomotion patterns, also called body bends

**Video S2. The Locomotion Pattern of *nca(lf)* Mutants**

A *nca-2(gk5);nca-1(gk9)* mutant animal displays a locomotion deficit termed as “Fainter”. The animal was capable of moving in a sinusoidal fashion upon stimulation (by probing, shown in this movie; or to survive, e.g., lack of food in their habitat, not shown), but stopped after only a couple of body bends.

**Video S3. The Locomotion Pattern of *nca(gf)* Mutants**

*unc-77(hp102)*, *nca(gf)* animals display constitutive and exaggerated body bends that make them appear “coiling” during both forward and backward movements.

**Video S4. *hp102* Encodes a Gain-of-function Mutation in *nca-1***

A wild-type animal expressing *nca-1* genomic fragments harboring the *hp102* mutation displayed the exaggerated body bends similar to those by *unc-77(hp102)* mutants (Video S3).

Found at doi:10.1371/journal.pbio.0060055.sv004 (2.2 MB MOV).

**Video S5. Calcium Imaging of a HSN Cell Body in a Wild-Type Animal**

4 X real time. The cameleon signal was pseudo-colored based on the YFP/CFP ratio value, with red representing the highest value. Each pink flash reflected the peak of one calcium spike.

**Video S6. The Locomotion Pattern of *unc-80* Mutants**

A *unc-80(e1272)* mutant animal displayed the same fainter locomotion deficit as *nca-2(gk5);nca-1(gk9)* mutants (Video S2).

**Video S7. *unc-80* Mutations do not Enhance the Phenotype of *nca(lf)* Mutants**

A *nca-2(gk5);nca-1(gk9);unc-80(e1272)* mutant animal displayed the same fainter locomotion deficit as in *nca(gk5);nca-1(gk9)* (Video S2) and in *unc-80(e1272)* (Video S6) mutants.

**Video S8. *unc-80* Mutations Suppress the Phenotype of *nca(gf)* Mutants**

A *unc-77(hp102); unc-80(e1272)* mutant animal displayed the same fainter phenotype as in *unc-80(e1272)* mutants (Video S6).

**Video S9. *F25C8.3* Rescues the Locomotion Deficit of *unc-80* Mutants**

A *unc-80(e1272)* mutant animal carrying an extrachromosomal array that consisted of genomic fragments covering the *unc-80* locus displayed continuous locomotion, and did not ‘faint’.

**Video S10. *F25C8.3* Reverts the *unc-80* Mutation-Mediated Suppression of *nca(gf)***

A *unc-77(hp102); unc-80(e1272)* mutant animal carrying an extrachromosomal array that consisted of genomic fragments covering the *unc-80* locus displayed continuous and exaggerated sinusoidal (coiling) locomotion pattern, similar to that of *unc-77(hp102)* mutants (Video S3).

**Video S11. NCA-1 Functions in Neurons**

A *nca-2(gk5); nca-1(gk9)* mutant animal carrying an extrachromosomal array expressing NCA-1 Driven by a pan-neuronal promoter displayed continuous and sinusoidal locomotion, and did not ‘faint.’

**Video S12. UNC-80 Functions in Neurons**

A *unc-80(e1272)* mutant animal carrying an extrachromosomal array expressing UNC-80 driven by a pan-neuronal promoter displayed continuous and sinusoidal locomotion, and did not ‘faint.’

**Video S13. *hp102*-Equivalent Mutation in Mouse NALCN Functions as NCA(gf) in *C. elegans***

A wild-type animal carrying an extrachromosomal array that expresses mouse NALCN(R329Q) driven by a pan-neuronal promoter displayed exaggerated body bends, which is characteristic of *hp102* and *e625* animals.

**Video S14. The Locomotion Pattern of *egl-19(ad695)* Gain-of-Function Mutants**

The mutant animals were hyperactive and egg-laying constitutive, but are behaviorally distinguishable from the “coiling” *unc-77(hp102)* mutants (Video S3).

**Supplemental figure legends:**

**Figure S1. *hp102* Mutants Affects Locomotion, Active Zone Marker Distribution at NMJs, and Egg-Laying Behavior, Which Were Fully Suppressed by *unc-80* Mutations**

(A) Images of the body morphology of *nca(gf)*, *nca(lf)*, *unc-80*, and *nca(gf);unc-80* mutants. The coiling position of *nca(gf)* was fully restored in *nca(gf);unc-80* mutants.

(B) Active zone marker morphology of DD GABAergic synapses in L2 larvae of wild-type, *hp102*, *unc-80*, and *nca-1(hp102); unc-80* animals, visualized by SYD-2::GFP (*hpIs3*). Inserts are magnified views of regions marked by the dotted line. *nca(gf)* mutants showed abnormal clustering (arrowheads) and gaps between active zone marker puncta. This defect was fully suppressed in *nca(gf);unc-80* mutants.

(C) Quantification of the average number (*n* = 10 for each strain) for *hpIs3* (~1/3 of dorsal cord region), where *nca(gf);unc-80* mutants showed wild-type level puncta.

(D) Morphology of HSN synapses visualized by an active zone marker *wyIs12* also showed abnormal clustering in *nca(gf) hp102* mutants (bottom panels) compared to wild-type animals (top panels). This phenotype was rescued in *nca(gf);unc-80* mutants.

(E and F) *nca(gf) hp102* mutants display constitutive egg-laying, resulting in fewer eggs (E, the total number of eggs) and younger eggs (F, % of eggs eight cells or younger) retained in uterus of *hp102* animals compared to wild-type animals. *n* = 15, the number of eggs was counted from animals 24-h post L4 larval stage. Error bar: SEM. Statistic comparisons were performed against the wild-type dataset using the Tukey-Kramer multiple comparison test. **p* < 0.01, ***p* < 0.001, Scale bar: 5μm.

**Figure S2. HSN Cell Bodies Autonomously Generated Calcuim Spikes Independent of Presynaptic Input Under the Assay Condition (10 mM HEPES, pH 7.1)**

A manuscript describing this work has been submitted. We presented here only one piece of supporting evidence. In *unc-13(e51)* mutants, where synaptic transmission is severely abolished, HSN cell bodies were capable of generating trains of calcium spikes. (A) Representative cameleon trace displayed by HSN cell bodies of *unc-13(e51)* mutants as in Figure 3.  (B) Calcium spike frequencies of HSN cell bodies in wild-type (wt) and *unc-13(e51)* mutants showed no statistically difference by the Kolmogorov-Smirnov rank test (*p* > 0.1). The number at the bottom of each bar represented the number of animals examined.

**Figure S3. Raw Traces of YFP and CFP Recorded in HSN Cell Bodies and Synapses**

The left panel, top two lines, show raw YFP and CFP traces for HSN cell body recordings shown in Figure 3. The *x-*axis represents the recording time in seconds, and the *y-*axis represents fluorescent intensity in arbitrary units of pixel intensity. The ratio between YFP and CFP fluorescent signals at each time point was plotted against the same *x-*axis, resulting in a third trace that represents the YFP/CFP ratio change.  Calcium spikes display a characteristic asymmetric shape, with a fast, linear rising phase followed by a slower, exponential decaying phase (arrows); whereas peaks due to noise, or random fluorescent ratio fluctuations, typically show a symmetric shape, with linear rising and decaying phases (arrowheads). Reciprocal changes in YFP and CFP intensity were observed for many ratio peaks (examples were shown by dashed lines), though reciprocity was sometimes obscured by motion artifacts caused by movement of the neurons during egg-laying.  Despite the fluctuations in the absolute YFP and CFP intensity levels over the recording period, the YFP/CFP ratio-metric trace revealed similar ratio changes for most calcium spikes in wild-type animals, suggesting that the calcium spike size is insensitive to fluorescent baseline changes and accurately reflects changes in calcium concentration. The right panel shows raw YFP and CFP traces for HSN synaptic trace shown in Figure 3.

**Figure S4. UNC-80 Encodes a Highly Conserved Novel Protein**

**(**A) A schematic representation of the gene structure of *unc-80 (F25C8.3)* adapted from the Wormbase. The exons are shown as pink boxes. The genetic lesions of *unc-80* alleles (*e1069*, *e1272*, and *hp369*) are shown. (B) Protein structure and similarity of UNC-80 family members. *C.e.*: *Caenorhabditis. elegans*; *D.m.:* *Drosophila melanogaster*; *R.n.:* *Rattus norvagicus*; *M.m.: Mus musculus*; *H.s*.: *Homo sapiens*.

**Figure S5. NCA-1 Functions Cell Autonomously in Neurons to Regulate Active Zone Morphology**

Active zone morphology was examined using the SYD-2::GFP marker *hpIs3* in wild-type and *nca(gf)* respectively. In wild-type animals, SYD-2::GFP puncta are round and regularly spaced, whereas *nca(gf)* animals show regions lacking puncta, as well as clustering of puncta. Genomic DNAs containing the *nca-1* gene, or expression of NCA-1 from a GABAergic neuron-specific promoter, restored the *hpIs3* phenotype of *nca(gf)* to wild-type morphology. Inserts show a higher magnification of the region underlined by the dotted line. Scale bar: 5 μm.

**Figure S6. NCA-1 and UNC-80 Are Enriched at Nonsynaptic Regions**

**(**A) Wild-type animals were co-stained with anti-NCA-1 antibody (red) and either anti-SNB-1 or anti-SAD-1 antibodies (green). (B) *unc-80; hpIs98* (UNC-80::RFP) animals were co-stained with anti-RFP antibody (red) and either anti-SNB-1 or anti-SAD-1 antibodies (green). Poor colocalization was observed in all cases. Scale bar = 5 μm.

**Figure S7. UNC-80::RFP Restores Localization of NCA-1 in *unc-80* Mutants**

(A) UNC-80 regulates NCA-1 protein localization. Wild-type and *unc-80* animals were stained with anti-NCA-1 (red) and anti-UNC-10 (green) antibodies. Punctate NCA-1 staining pattern along the ventral and dorsal nerve cords was observed in wild-type but diminished in *unc-80* animals. UNC-10 staining pattern was similar between wild-type and *unc-80* mutant animals. The expression of UNC-80::RFP from its own promoter (*hpIs98*) restored the punctate expression pattern of NCA-1 along the nerve cords.

(B) *unc-80* mutations do not affect the transcript level of *nca-1*. Total RNA was isolated from mixed staged wild-type, *unc-80*(*e1069)* and *unc-80(e1272*) animals, and the transcript levels of *nca-1* were analyzed by RT-PCR (with a control reaction containing no template). *nca-1* transcript was present at similar levels in all strains. Scale bar: 5 μm.

**Figure S8. Endogenous UNC-79 Is Expressed in the Nervous System**

(A) An antibody against UNC-79 was used to stain wild-type (top panel) and *unc-79* animals (bottom panel). Specific staining was observed in the nerve ring of wild-type but not in *unc-79* mutant animals. *ns: non-specific staining. (B) The dorsal (top panel) and ventral (bottom panel) nerve cords of wild-type animals stained with anti-UNC-79 antibody showed punctate staining pattern.

**Figure S9. UNC-79 Localization Depends on the Presence of UNC-80 and NCA**

Wild-type, *nca(gf)*, *nca(lf)*, *unc-80*, *nca(lf);unc-80*, and *nca(gf);unc-80* animals were simultaneously stained with anti-UNC-79 antibody. UNC-79 signals were detected in the nerve ring (NR) of wild-type and *nca(gf)* animals but disappeared in other mutants.

**Table S1. Summary of Phenotypes Displayed by *nca* and VGCC Single and Double Mutants**

*nca(lf)* or *unc-80* mutants in combination with *cca-1*, *egl-19* (*lf* and *gf*) and *unc-2* mutants display enhanced or additive locomotion defects than either single mutants. Similarly, *nca(gf)* mutants in *unc-2* and *egl-19*(*lf)* backgrounds lead to more severe or additive locomotion phenotypes. In particular, *egl-19(gf)* mutations do not suppress or improve the locomotion defects of *unc-80* and *nca(lf)* mutants. *nca(gf)* mutations do not improve the locomotion defects of *egl-19(lf)* or *unc-2(lf)* mutants.

This suggests that NCA/UNC-80 and VGCCs do not function in a linear pathway. Their genetic interactions are most consistent with these two types of channels regulating different aspects of neuronal excitation.

**SUPPLEMENTAL REFERENCES**

1. Altun-Gultekin, Z., Andachi, Y., Tsalik, E. L., Pilgrim, D., Kohara, Y., and Hobert, O. (2001). A regulatory cascade of three homeobox genes, *ceh-10*, *ttx-3* and *ceh-23*, controls cell fate specification of a defined interneuron class in *C. elegans*. Development 128, 1951-1969.

2. Hobert, O. (2002). PCR fusion-based approach to create reporter gene constructs for expression analysis in transgenic *C. elegans*. Biotechniques 32, 728-730.

3. Nagai, T., Ibata, K., Park, E. S., Kubota, M., Mikoshiba, K., and Miyawaki, A. (2002). A variant of yellow fluorescent protein with fast and efficient maturation for cell-biological applications. Nat Biotechnol 20, 87-90.

4. Kerr, R.A. Imaging the activity of neurons and muscles (June 2, 2006), WormBook, ed. The *C. elegans* Research Community, WormBook, doi/10.1895/wormbook.1.113.1, http://www.wormbook.org
