# Supplementary material for: A Putative Cation Channel, NCA-1, and a Novel Protein, UNC-80, Transmit Neuronal Activity in C. elegans
Source: PLoS Biol. 2008 Mar 11;6(3):e55. doi: 10.1371/journal.pbio.0060055 (PMC2265767; doi:10.1371/journal.pbio.0060055)
Supplement: Supplementary file 11 [file pbio.0060055.st001.doc]

**Table S1**: Behavioral phenotypes of double mutants between *unc-80, nca lf* and *nca gf* and VGCC mutants in *C. elegans*

|  | VGCC  type | Subunit | Behavioral Phenotypes | | | |
| --- | --- | --- | --- | --- | --- | --- |
|  |  |  | Single Mutant | Double with  ***unc-80* (fainter)** | Double with  ***nca lf* (fainter)** | Double with  ***nca gf* (coiler,**  **hyperactive**) |
| ***unc-2(ra612) lf*** | P/Q/N | α1 | weak kinker, slow | completely paralyzed | Same as left | Same as left |
| ***unc-36(ra612) lf*** | L | α2/δ | very sluggish,  almost paralyzed | completely paralyzed | Same as left | Same as left |
| ***egl-19(n582) lf*** | L | α1 | sluggish, long,  Egl-d | completely paralyzed  Egl-d | completely paralyzed | Intermediate  locomotion and  length,Egl-d, coiler |
| ***egl-19(ad695) gf*** | L | α1 | hyperactive,  short, Egl-c | short fainter, Egl-c | N/A | N/A |
| ***cca-1(ad1650) lf*** | T | α1 | no locomotion  defect | fainter | Same as left | coiler |

- *lf*: loss-of-function *gf:* gain-of-function

- N/A: not available

- Egl-c: egg-laying constitutive

- Egl-d: egg-laying defective
